# Polymorphism and Structural Distortions of Mixed-Metal Oxide Photocatalysts Constructed with *α*-U_3_O_8_ Types of Layers

**DOI:** 10.3390/cryst7050145

**Published:** 2017

**Authors:** Nacole King, Jonathan Boltersdorf, Paul A. Maggard, Winnie Wong-Ng

**Affiliations:** 1 National Institute for Standards and Technology, Materials Measurement Science Division, 100 Bureau Drive, Gaithersburg, MD 20899, USA; 2 United States Army Research Laboratory, Sensors and Electron Devices Directorate, Adelphi, MD 20783, USA; 3 North Carolina State University, Department of Chemistry, Raleigh, NC 27695, USA

**Keywords:** photocatalysis, mixed-metal oxide, structural distortion

## Abstract

A series of mixed-metal oxide structures based on the stacking of *α*-U_3_O_8_ type pentagonal bipyramid layers have been investigated for symmetry lowering distortions and photocatalytic activity. The family of structures contains the general composition ${{\text{A}}^{m+}}_{\left((n+1)/\text{m}\right)}{\text{B}}_{\left(3n+1\right)}{\text{O}}_{\left(8n+3\right)}$ (e.g., A = Ag, Bi, Ca, Cu, Ce, Dy, Eu, Gd K, La, Nd, Pb, Pr, Sr, Y; B = Nb, Ta; m=1-3; n=1, 1.5, 2), and the edge-shared BO_7_ pentagonal pyramid single, double, and/or triple layers are differentiated by the average thickness, (i.e., 1 ≤ *n* ≤ 2), of the BO_7_ layers and the local coordination environment of the “A” site cations. Temperature dependent polymorphism has been investigated for structures containing single layered (n=1) monovalent (m=1) “A” site cations (e.g., Ag_2_Nb_4_O_11_, Na_2_Nb_4_O_11_, and Cu_2_Ta_4_O_11_). Furthermore, symmetry lowering distortions were observed for the Pb ion-exchange synthesis of Ag_2_Ta_4_O_11_ to yield PbTa_4_O_11_. Several members within the subset of the family have been constructed with optical and electronic properties that are suitable for the conversion of solar energy to chemical fuels via water splitting.

## 1. Introduction

Many properties and applications of inorganic materials are derived from a small number of reported structure types. Structural families of metal-oxides that have been commonly explored in the literature are ABO_3_ pervoskites [1–5], layered Ruddelsden-Popper pervoskites [6–8], and spinel structures [9–11]. Metal-oxide materials can also be classified by their properties that can include superconductors [12–15], transparent conducting oxides [16–20], and photocatalysts [21–26]. Variations of compositions within homologous families of structures exhibit important structural transformations (i.e., structural distortions, polymorphism) which impact their optical properties (i.e., range of light absorption for photocatalysis). A family of structures based on the stacking of *α*-U_3_O_8_ type layers constructed with pentagonal bipyramids is the focus of this review, wherein condensed MO_7_ (M = Nb, Ta) polyhedra have been found to underlie interesting new polymorphism, structural distortions, and activity for photocatalytic water splitting.

The crystal structure of U_3_O_8_ was first proposed by Zachariasen [27], further investigations of the single crystal and neutron diffraction confirmed the orthorhombic crystal structure of U_3_O_8_ [28–30]. At room temperature two crystalline forms of U_3_O_8_ can be synthesized. The *α*-U_3_O_8_ phase is the form obtained under typical synthetic conditions; however, under special circumstances the β-U_3_O_8_ form can be crystalized [31]. The *α*-U_3_O_8_ structure undergoes a phase transition from an orthorhombic unit cell to a hexagonal unit cell at ≈210 °C [32–34]. A polysomatic family of structures with the general composition ${{\text{A}}^{m+}}_{\left((n+1)/m\right)}{\text{B}}_{\left(3n+1\right)}{\text{O}}_{\left(8n+3\right)}$ (e.g., A = Ag, Bi, Ca, Cu, Ce, Dy, Eu, Gd, K, La, Nd, Pb, Pr, Sr, Y; B = Nb, Ta; n=1,1.5,2; m=1–3) have been constructed with *α*-U_3_O_8_ type layers comprised of mixed six-, seven-, and eight-coordination sites [35]. Synthesis and structural investigations of these systems were first described in detail by Jahnberg [36–38]. Furthermore, several of the structures in the system display symmetry lowering distortions, and unique optical and electronic properties that are viable for the direct conversion of solar energy into chemical fuels.

## 2. *α*-U_3_O_8_ Type Structural Layers

**Figure 1.**
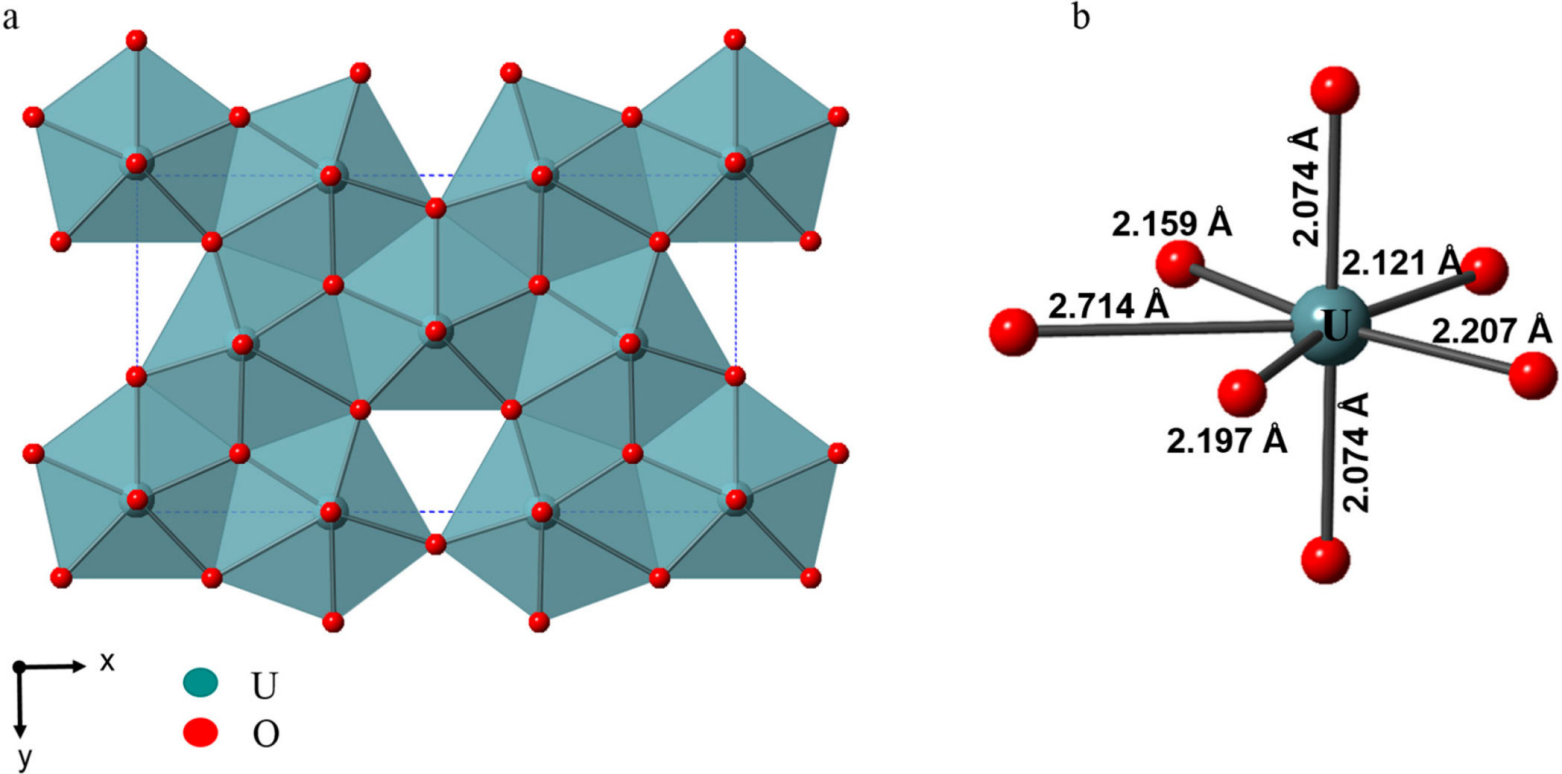
(**a**) Polyhedral view of the crystal structure of *α*-U_3_O_8_ showing the edge-shared UO_7_ pentagonal bipyramid layer in the (001) plane and (**b**) the local pentagonal bipyramidal coordination environment of a U cation annotated with interatomic distances.

**Figure 2.**
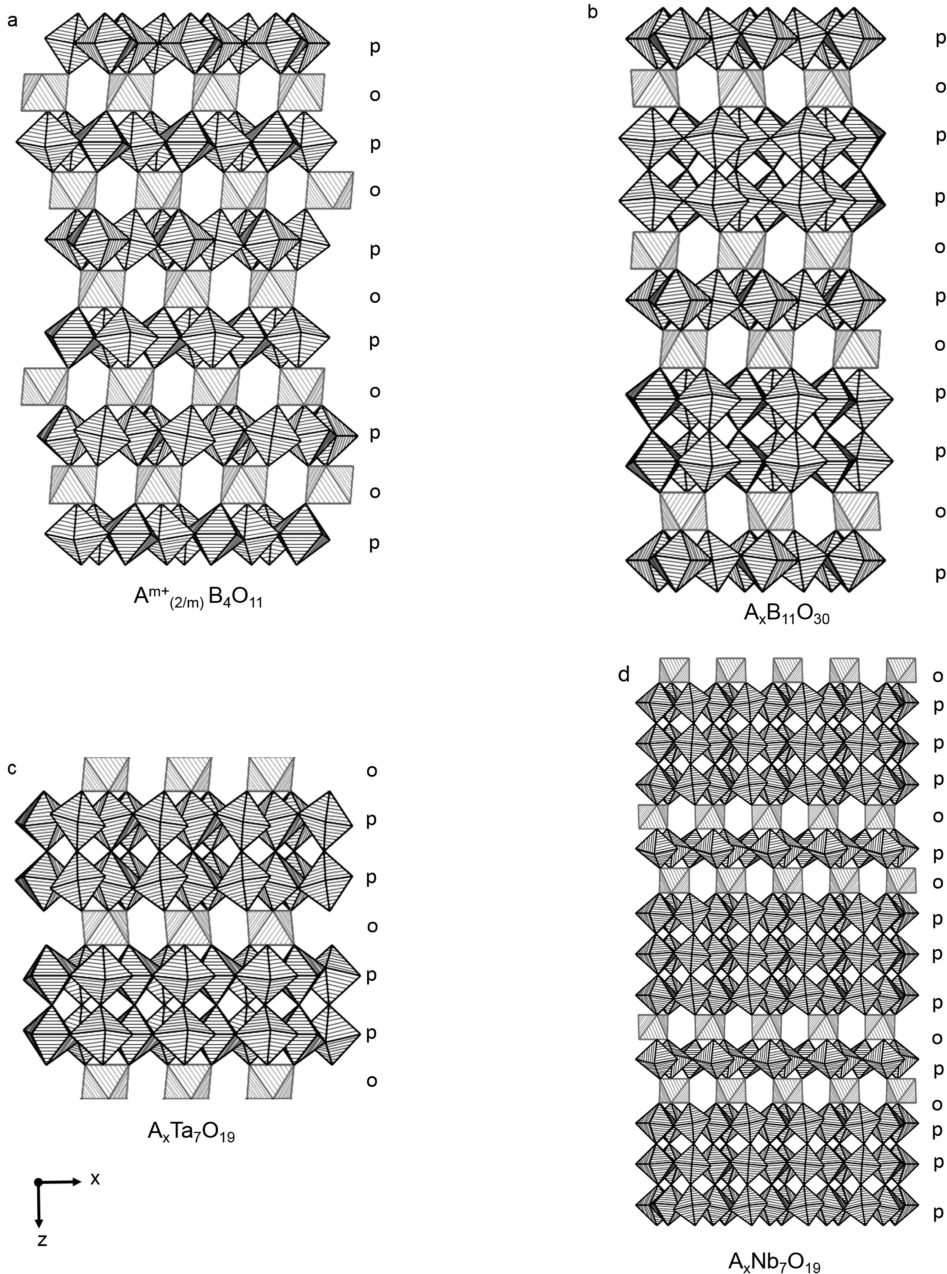
The crystal structures constructed from *α*-U_3_O_8_ type layers with the general composition ${{\text{A}}^{\text{m}+}}_{\left((\text{n}+1)/\text{m}\right)}{\text{B}}_{\left(3\text{n}+1\right)}{\text{O}}_{\left(8\text{n}+3\right)}$ (**a**) single; (**b**) alternating single and double; (**c**) double; and (**d**) alternating single and triple layers of edge-shared pentagonal bipyramid polyhedra. These layers alternate with layers of isolated octahedra along the (001) direction. The grey (o) and black (p) striped polyhedra represent BO_6_ octahedral and BO_7_ layers, respectively.

The orthorhombic crystal structure of *α*-U_3_O_8_ (space group *Amm*2 (#38) *a* = 4.14(8) Å, *b* = 11.96(6) Å, *c* = 6.71(7) Å) consists of layers of edge-shared pentagonal bipyramids along the (110) direction [30]. In each layer a maximum of four edges can be shared, leaving one unshared edge per polyhedron [36]. An isolated UO_7_ pentagonal bipyramid layer is built from U_3_O_5+6/2_ layers, where 6/2 are the six shared apical oxygen atoms between alternating layers, thereby yielding the U_3_O_8_ chemical composition [37]. The average interatomic distances between six nearby O atoms for U1 and U2 are between 2.07 Å–2.23 Å, with each U atom having a longer interatomic distance for a seventh O atom 2.44 Å (U1) and 2.71 Å (U2). An edge shared layer of UO_7_ polyhedra and an isolated UO_7_ pentagonal bipyramid labeled with average atomic distances and a single edge-shared polyhedra are shown in Figure 1a,b, respectively. This edge-shared pentagonal bipyramid layer is the principle building block for a polysomatic family of structures with the general composition ${{\text{A}}^{m+}}_{\left((n+1)/m\right)}{\text{B}}_{\left(3n+1\right)}{\text{O}}_{\left(8n+3\right)}$ (e.g., A = Ag, Bi, Ca, Cu, Ce, Dy, Eu, Gd, K, La, Nd, Pb, Pr, Sr, Y; B = Nb, Ta; *n* = 1, 1.5, 2; *m* = 1–3). These ternary mixed-metal oxides are constructed from pentagonal bipyramidal layers, similar to the layers observed in *α*-U_3_O_8_, where n defines the average thickness (1 ≤ *n* ≤ 2) of the BO_7_ layers [35]. Furthermore, these structures are comprised of either 2, 7, or 8 coordination sites for the A-site cation, and layers of single, double, and triple layers of edge-shared pentagonal bipyramids. The crystal structures are comprised of edge-shared pentagonal bipyramid polyhedra that stack in single, double, and triple layers, as shown in Figure 2.

### 2.1. Single Layers

**Figure 3.**
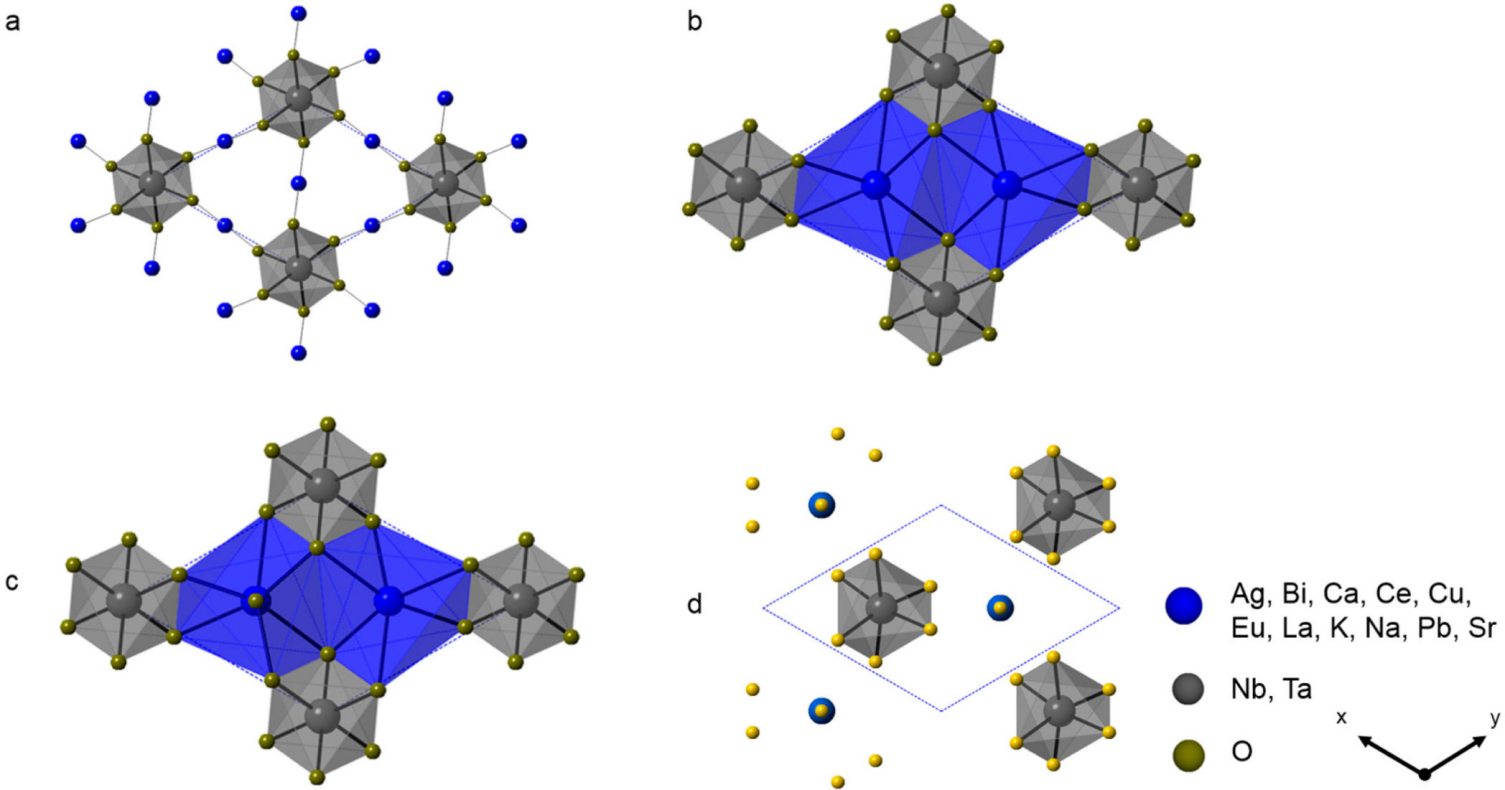
Polyhedral view of the MO_6_ layer (M = Nb or Ta), illustrating the occurrence of (**a**) 2- (**b**) 6- (**c**) 7- (**d**) 8-fold coordination number for the A site cations that stack in single, double, and triple (Nb/Ta)O_7_ layers.

Crystal structures comprised of single pentagonal bipyramidal layers have been observed for the subset of metal oxides with the general composition ${{\text{A}}^{m+}}_{\left(2/m\right)}$ B_4_O_11_ (A = Ag, Ca, Cu, K, Na, Pb, Sr; B = Nb, Ta; m=1,2) [37–39]. These layers of MO_7_ polyhedra alternate with layers of MO_6_ octahedra in a p–o–p–o (o = octahedra, p = pentagonal bipyramid) pattern, as shown in Figure 2a. Individual structural views of each type of layer are shown in Figure 3a–d. The apical O atoms of the single layers of edge-shared Nb/Ta pentagonal bipyramids form the intervening coordination environments (from above and below) for the layers of Nb/Ta octahedra and A-site cations. Each BO_6_ octahedral environment can accommodate either three (m=2) or six (m=1) A-site cations that are 2, 6, 7, or 8-coordinate depending on their oxidation state, size, and coordination preference. Linearly coordinated cations have solely been observed for Cu-containing compounds (e.g., Cu_2_Ta_4_O_11_) where Cu vacancies are found to occur over 33% of the available Cu sites [40]. There are six nearest neighbor O atoms for coordination to the A-site cations in the Ag/K tantalates and the Ag/Na niobates. The coordination number for the Na/Pb cations in Na_2_Ta_4_O_11_ and PbTa_4_O_11_ is 7, owing to an increase of a single nearest neighbor O atom in comparison to Na_2_Nb_4_O_11_. The crystal structure for CaTa_4_O_11_ is shown in Figure 3d, where the Ca^2+^ and Sr^2+^ cations are surrounded by eight nearest neighbor O atoms. Alternating octahedral and pentagonal bipyramidal layers are mirror images of one another; thus, upper and lower faces of the BO_6_ (B = Nb, Ta) octahedron cavity are formed from three oxygen atoms in the layer above and from three oxygen atoms in the layer below [36]. The layers are bridged through the apical vertices of BO_7_ (B = Nb, Ta) pentagonal bipyramids that are perpendicular to BO_6_ (B = Nb, Ta) octahedral layers. The interatomic distances for 7-coordinate Nb/Ta polyhedra are included in Table S1.

The rhombohedral $R\bar{3}c$ space group is observed for members of the ${{\text{A}}^{m+}}_{\left(2/m\right)}$B_4_O_11_ subset of structures where the A-site cation is monovalent (*m* = 1). Although the symmetry of the compositions Ag_2_Nb_4_O_11_, Ag_2_Ta_4_O_11_, Cu_2_Ta_4_O_11_, Na_2_Nb_4_O_11_, Na_2_Ta_4_O_11_, and K_2_Ta_4_O_11_ are similar, the coordination geometries of the A-site cations vary as previously mentioned. Babaryk et al., reported increasing the calculated polyhedral volume of MO_x_ (*x* = 6–8) leads to successful transformations from centrosymmetric to noncentrosymmetric space groups [41]. However, Na_2_Nb_4_O_11_ and Ag_2_Nb_4_O_11_ are not polar at room temperature, and are classified in a rhomobohedral R3c and monoclinic C2/c space groups, respectively [42,43]. Table S2 in the Supporting Information indicates three general trends for extended structures with the composition A^*m*+^_(2/*m*)_B_4_O_11_: (1) an increase in the “A” site coordination number is directly correlated to decreasing *c* lattice constant parameters; (2) members where *m* = 1 belong to centrosymmetric nonpolar space groups which contain an inversion center; and (3) members where *m* = 2 belong to noncentrosymmetric polar space that do not contain an inversion center. Symmetry-lowering structural distortions have been reported for the monovalent A-site niobate cations Na and Ag, as well as the monovalent “A”site Cu cation in the tantalate structures, which will be further discussed in a separate section of this review.

### 2.2. Alternating Single and Double Layers

The Cu_5_Ta_11_O_30_ and Pr_2_Nb_11_O_30_ compounds are members of the subgroup of structures that are comprised of alternating layers of single and double (Nb/Ta)O_7_ pentagonal bipyramids [44–46] shown in Figure 2b. The members of this subset of structures are arranged in a stacking scheme constructed with octahedral (o) and pentagonal bipyramid (p) layers in a p-o-p-p-o order. The edge-shared pentagonal bipyramid layers and layers of isolated BO_6_ octahedra surrounded by 2- or 8-coordinated Cu (I) or Pr cations in Figure 3a,d, respectively. As previously observed for Cu-containing structures comprised of layers of edge-shared pentagonal bipyramids, the Cu atoms in Cu_5_Ta_11_O_30_ are also linearly coordinated and contain Cu vacancies, where 5/6 of the Cu sites are occupied[44,47]. Furthermore, the Cu-containing phase with the theoretical composition Cu_7_Ta_15_O_41_ was observed as a side product during the Cu_5_Ta_11_O_30_ phase analysis study by Jahnberg [48] and indexed on a hexagonal unit cell in the P6_3_/m space group. The Pr_2_Nb_11_O_30_ compound is structurally related to Cu_5_Ta_11_O_30_ and is the only known example that appears to be partially reduced (i.e., ≈9.1% Nb^4+^ to ≈90.9% Nb^5+^) within the Jahnberg structural family [49]. The structure is comprised of one symmetry unique Pr (III) cation coordinated to eight near-neighbor O atoms and three symmetry unique Nb cations, where Nb1 and Nb2 are edge shared NbO_7_ polyhedra, and Nb3 is octahedrally coordinated to surrounding O atoms. Selected crystallographic information for Cu_5_Ta_11_O_30_, Cu_7_Ta_15_O_41_, and Pr_2_Nb_11_O_30_ are included in Table S3 in the Supporting Information.

### 2.3. Double Layers

The subset of structures composed exclusively with double layers of edge-shared TaO_7_ (B = Ta) pentagonal bipyramids occur with the general formula ${\text{A}}^{m+}((n+1)/m)$ Ta_7_O_19_ (A = Bi, Cu, Ce, Dy, Eu, Gd, La, Nd, Sm, Y; *m* = 1, 3; *n* = 1.5, 2) [35,40,46,50–53]. Members of this subset are constructed with octahedra (o) and pentagonal bipyramids (p) in the following stacking pattern p-p-o-p-p-o as shown in Figure 2c. Similar to the A-site cations in the sub-group of structures composed of single layers and alternating layers of single double pentagonal bipyramids, Cu-containing cations (m=1) within the branch of structures composed of double layers of TaO_7_ pentagonal bipyramids are linearly coordinated to O atoms as shown in Figure 3a. With the exception of Cu, the A-site cations, i.e., Bi, Ce, Eu, Dy, La, Y (m=3), are coordinated to eight nearby O atoms within the layer containing isolated TaO_6_ octahedra. Linearly coordinated Cu and 8-coordinated A-site cations are shown in Figure 3a. Similar to the single layered structures, the double-layered structure is constructed of mirrored images of alternating octahedral and pentagonal bipyramidal layers bridged through the apical vertices of the TaO_7_. The interatomic distances for TaO_7_ polyhedra of selected compounds are listed in Table S4.

The members constructed with double layers of TaO7 pentagonal bipyramids can be classified to the hexagonal crystal systems, (i.e., P6_3_/m, $\text{P}\bar{6}\text{c}2$, and P6_3_/mcm) as listed in Table S4. Very early investigations by Chretien and Bodit found that the rare earth tantalates, ATa_7_O_19_ (A = Ce, Eu, Gd, La, Nd, Pr, Sm,) are isostructural to CeTa_7_O_19_ indexed on a tetragonal unit cell, as found using powder X-ray diffraction [54,55]. Further powder X-ray and single crystal investigations by Rossel and Gatehouse concluded CeTa_7_O_19_ crystalizes in the hexagonal system, and includes the other known rare earth tantalates. [50,53,56]. Similarily, YTa_7_O_19_ was indexed on a hexagonal unit cell, space group determination among the P6_3_/m, $\text{P}\bar{6}\text{c}2$, and P6_3_/mcm was not distinguishable [53]. Niobium containing chemical compositions with the general formula ${\text{A}}^{m+}((n+1)/m)$Nb_7_O_19_ cations have also been found, and are composed of triple NbO_7_ layers, as described below.

### 2.4. Alternating Single and Triple Layers

**Figure 4.**
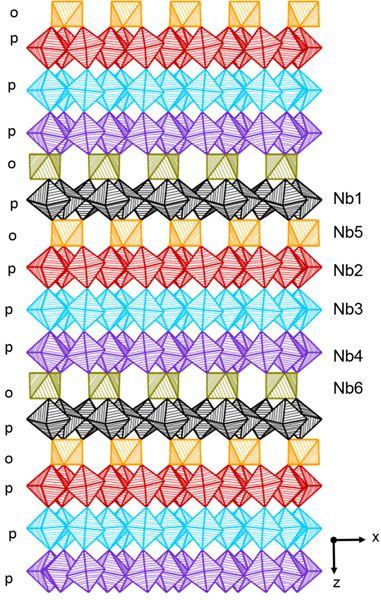
The crystal structure LaNb_7_O_19_, with labels for the symmetry inequivalent Nb cations in each layer. The layers of NbO_7_ pentagonal bipyramids are shown in black (Nb1), red (Nb2), blue (Nb3), and purple (Nb4) stripped polyhedra. The NbO_6_ octahedra are shown in orange (Nb5) and gold (Nb6) striped polyhedra.

Hofmann and Gruehn synthesized and reported a subset of lanthanide rare earth structures with the general composition ${{\text{A}}^{m+}}_{\left((n+1)/m\right)}$Nb_7_O_19_ (A = Ce, La; m=3; n=2) [57]. These rare earth mixed-metal oxide structures are comprised of triple NbO_7_ pentagonal bipyramid layers alternating with 8-coordinated La and Nb octahedra, as shown in Figure 2d. A single crystal investigation of LaNb_7_O_19_ confirmed that it crystallizes in the trigonal space group, *P3* [57]. Its structure is composed of eight-coordinated La^3+^ cations that form distorted square antiprisms and Nb cations in distorted octahedra which are linked together via corner shared O atoms as shown in Figure 3. The *α*-U_3_O_8_-type layers of NbO_7_ are mirror images of one another and alternate with the following stacking scheme p-o-p-p-p-o (p = pentagonal bipyramid and o = octahedra), as shown in Figure 4. Each type of niobate layer is constructed from its own symmetry unique Nb cation, where Nb1–4 are layers of pentagonal bipyramids and Nb5 and Nb6 are the layers of octahedra. The structures LaNb_7_O_19_ and CeNb_7_O_19_ are the first example of a trigonal member within the family of structures. A single crystal investigation was not carried out for CeNb_7_O_19_, the assumption of an isotype with LaNb_7_O_19_ is based on the consistency of the reflections from Guinean diagrams via powder X-ray diffraction [57]. From the investigation, it cannot be ruled out that Ce^4+^ may occupy the La^3+^ and Nb^5+^ sites. Furthermore, powder X-ray investigation for NdNb_7_O_19_ and PrNb_7_O_19_ were reported and the structural information is provided in Table S3 in the Supporting Information [46,58].

## 3. Structural Distortion and Polymorphism

Symmetry-lowering distortions have been observed for several of the Nb- and Ta-containing structures constructed with *α*-U_3_O_8_ type pentagonal bipyramid layers. For example, structures composed of single MO_7_ pentagonal bipyramid layers (e.g., Ag_2_Nb_4_O_11_, Cu_2_Ta_4_O_11_, Na_2_Nb_4_O_11_, PbTa_4_O_11_) have exhibited structural distortions under various conditions (e.g., as a function of temperature, composition, etc.). Second-order Jahn-Teller (SOJT) effects by d^0^ cations in octahedral coordination environments can be the driving force for structural distortions and symmetry lowering transitions, such as leading to noncentrosymmetric structures [59]. The SOJT distortions have typically been found for octahedral d^0^ cations with high valency, with the magnitude of the distortion increasing in the following order: Zr^4+^ < Ta^5+^ < Nb^5+^ < W^6+^ < V^5+^ < Mo^6+^ [59]. The magnitude of the distortions are expected to increase as the energy separation between the metal-based t_2g_ *d*-orbitals and the non-bonding O 2p states decrease, owing to a greater mixing of the occupied (O based) and unoccupied (M based) crystal orbitals [60]. Another theory described by Kunz and Brown finds that the directions of the displacements of the d^0^ cations are not primarily determined from the electronic SOJT effect [61], but rather the cation displacement is influenced more by bond network stresses (e.g., asymmetry in the bond network from the nearest neighbors). In addition, cation-cation repulsions from near-neighbor cations that share edges or faces also increase bond network stresses.

### 3.1. Niobates

Symmetry lowering transitions that are a function of temperature have only been reported for the niobium-containing structures Ag_2_Nb_4_O_11_ and Na_2_Nb_4_O_11_ [42,43]. The Ag_2_Nb_4_O_11_ phase transforms from the centrosymmetric $R\bar{3}c$ space group to the noncentrosymmetric R3c space group upon cooling to ≈127 °C. The driving force of this symmetry-lowering transition has been described to arise from the displacement of Ag and Nb cations within the pentagonal bipyramid and the octahedral layers [43]. The Ag and Nb cations are shifted out of the center of the polyhedra towards the octahedral faces of the distorted MO_6_ (M = Ag, Nb) polyhedra, yielding a dipole moment along the *c* axis [43]. The Nb^5+^ cations in pentagonal bipyramidal layers are displaced towards the apical O atoms in the polyhedra [43]. A second symmetry-lowering transition into the R3 space group is observed upon cooling to −73 °C and has been attributed to shifts in the equatorial O atoms of the NbO_7_ pentagonal bipyramids are found to occur [43].

By comparison, the symmetry lowering distortions of Na_2_Nb_4_O_11_ from $R\bar{3}c$ to C2/c upon cooling to ≈107 °C yields a nonpolar centrosymmetric structure [43,62]. The structural relationship of the phase transformation between the monoclinic and hexagonal-rhombohedral unit cells has been previously described [42,63]. The origin of the structural distortion is primarily related to the displacement of the Nb cation within the plane of the pentagonal bipyramidal layer [42]. Below the transition temperature, two crystallographically distinct Nb cations, (i.e., Nb1 and Nb2) are generated in the pentagonal bipyramidal layer [42]. The longest interatomic distance between Nb1-O4 increases, causing its polyhedron to further distort in the layer, while the Nb2 polyhedron becomes a more regular pentagonal bipyramid [42].

### 3.2. Tantalates

#### 3.2.1. Cu_2_Ta_4_O_11_

**Figure 5.**
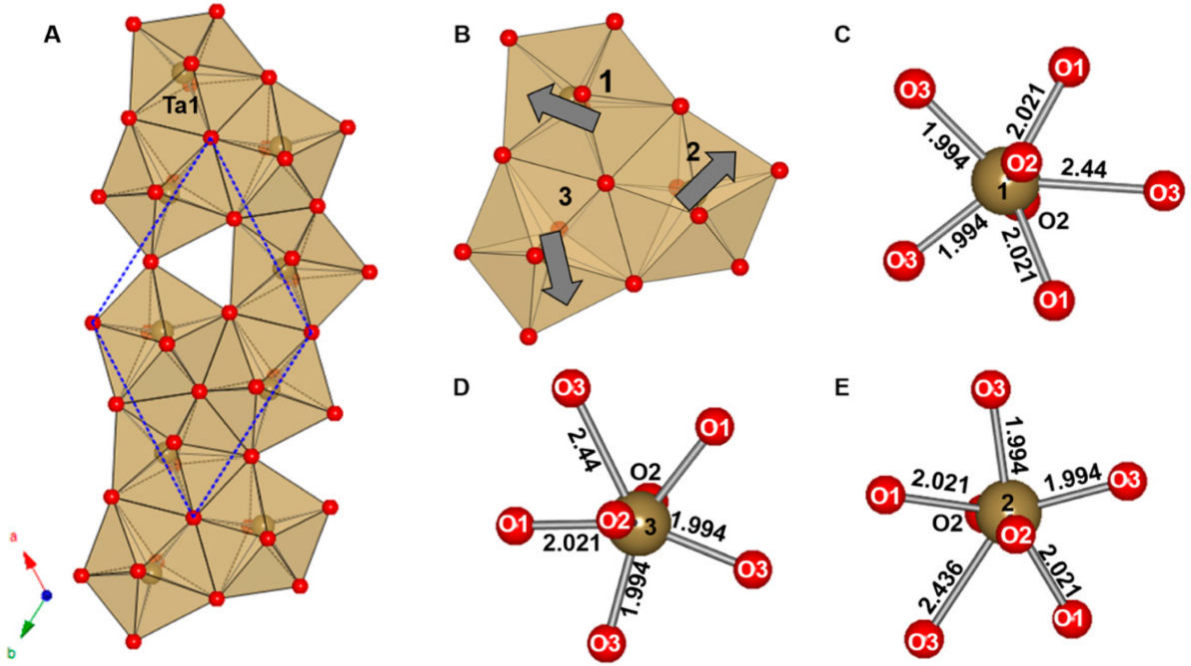
The crystal structure of a single edge-sharing TaO_7_ pentagonal bipyramid layer of the rhombohedral β-Cu_2_Ta_4_O_11_ (**A**) a view of the edge sharing of Ta cations along the *ab* plane with the unit cell outline as a blue dashed line (**B**) a smaller segment from the uppermost three Ta cations, where the grey arrows indicate the out-of-center displacement of Ta; and (**C**–**E**) local views of the coordination environments of the Ta cations labeled 1, 2, and 3, respectively. Reproduced with permission from [64], published by Elsevier, 2014.

**Figure 6.**
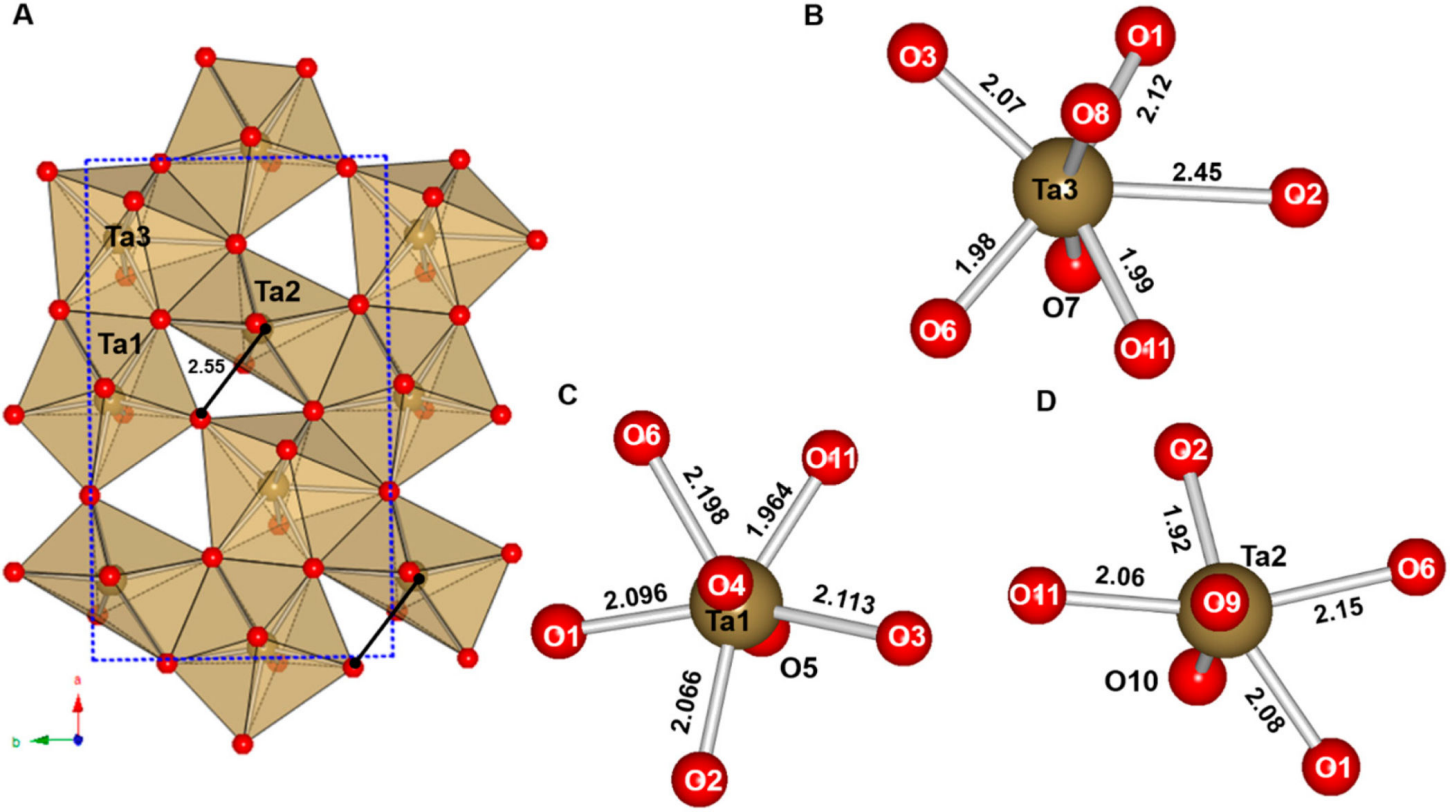
(**A**) Polyhedral view of a layer of edge-sharing TaO_7_ pentagonal bipyramids in the monoclinic *α*-Cu_2_Ta_4_O_11_, with the unit cell outline as a blue dashed line and the black line indicating the distortion of Ta2 into an octahedron; and (**B**–**D**) the local coordination environment of Ta3, Ta1, and Ta2, respectively. Reproduced with permission from [64], published by Elsevier, 2014.

The Cu_2_Ta_4_O_11_ structure occurs in two polymorphs, namely, the monoclinic *α*-Cu_2_Ta_4_O_11_ phase and the rhombohedral β-Cu_2_Ta_4_O_11_ [64,65], shown in Figures 5 and 6. At temperatures of −50 °C to room temperature, the monoclinic *α*-Cu_2_Ta_4_O_11_ phase is relatively stable. The rhombohedral β-Cu_2_Ta_4_O_11_ phase emerges upon heating the monoclinic *α*-Cu_2_Ta_4_O_11_ phase to between 250 °C and 550 °C [64]. This symmetry-lowering distortion is driven by the edge-shared Ta d^0^ cations that form the single pentagonal bipyramidal layers [64]. The direction of the out-of-center displacement of the Ta cations are towards the vertices of the polyhedra not shared by neighboring Ta cations, as illustrated in Figures 5 and 6 for the distorted and non-distorted structures, respectively [64]. Thus, it appears that the repulsion from nearest-neighbor cations directs the out-of-center displacement of Ta d^0^ cations towards an edge of the polyhedron that is not shared by a neighboring Ta cation within the layer [64]. The longer interatomic distance between Ta2-O2 in the pentagonal bipyramidal layer increases from 2.44 Å to 2.55 Å; thus, the Ta2 cation, coordinated to 7 O atoms in a pentagonal bipyramid, is transformed into a highly-distorted TaO_6_ octahedron within the tantalate layer [64].

An explanation of the phase transition observed in the rhombohedral β-Cu_2_Ta_4_O_11_ can be suggested from bond length arguments. The longer Ta-O interatomic distances observed in the TaO_6_ and TaO_7_ polyhedra in Cu_2_Ta_4_O_11_ are comparable to the Nb-O distances in Na_2_Nb_4_O_11_ and Ag_2_Nb_4_O_11_. Thus, these polyhedra are more flexible to accommodate an out-of-center displacement of the Ta/Nb cations. In the rhombohedral β-Cu_2_Ta_4_O_11_ structure, effects from cation-cation repulsion likely cause the nearest neighbor Ta 5d^0^ cations within three edge-shared TaO_7_ polyhedra to move out of the center of the polyhedra. The TaO_7_ pentagonal bipyramid layer distorts into a layer containing TaO_6_ and TaO_7_ polyhedra, where Ta3 and Ta1 maintain the TaO_7_ pentagonal bipyramid coordination environment and Ta2 centers a highly-distorted TaO_6_ octahedron [64]. The monoclinic *α*-Cu_2_Ta_4_O_11_ structure contains fewer edges that are shared as the Ta2-O2 interatomic distance lengthens to ≈2.55(3) Å, an increase of ≈0.13 Å as compared to the rhombohedral β-Cu_2_Ta_4_O_11_ structure [64]. Structural distortions to lower symmetry have not been observed for Ag_2_Ta_4_O_11_, which could be due to the slightly larger interatomic distances in the equatorial plane of edge-shared pentagonal bipyramids for Ta1-O3 in Cu_2_Ta_4_O_11_ as compared to those in Ag_2_Ta_4_O_11_, at 2.45(8) Å and 2.398(2) Å, respectively [43]. The TaO_6_ octahedron are also relatively larger, in the range of 1.92(7) Å–2.20(6) Å, as compared to those in Ag_2_Ta_4_O_11_ at 1.9845(5) Å [43].

#### 3.2.2. PbTa_4_O_11_

**Figure 7.**
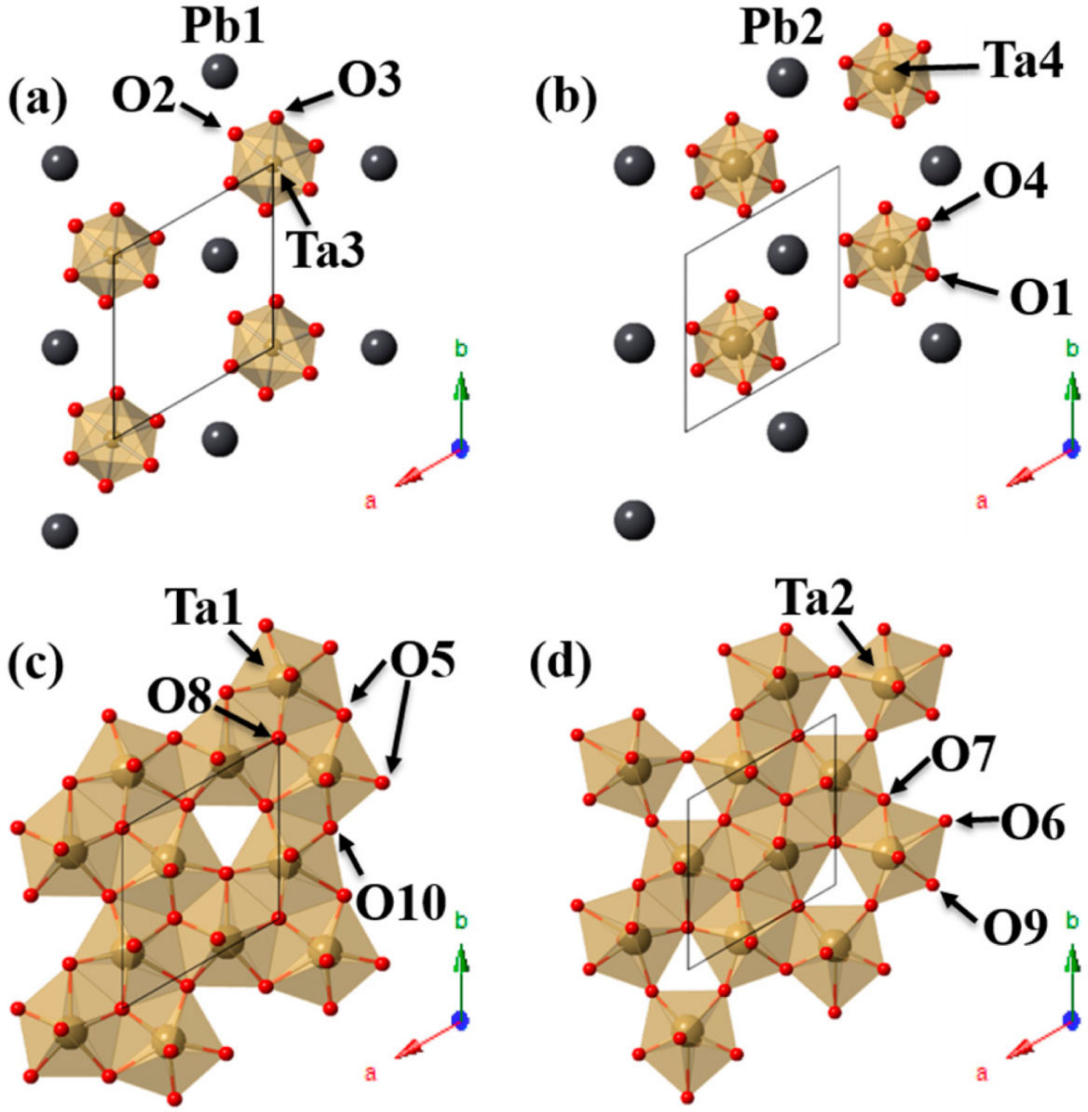
Polyhedral view of the four symmetry-unique layers of PbTa_4_O_11_ that are composed of alternating layers of (**a**–**b**) isolated TaO_6_ octahedra surrounded by Pb(II) cations and (**c**–**d**) edge-sharing TaO_7_ pentagonal bipyramid layers. The unit cell is outlined in black with tan-colored TaO_6_ and TaO_7_ polyhedra, gray spheres for Pb, and red spheres for oxygen. Reproduced with permission from [35], published by Elsevier, 2015.

Comparable to that described above for Cu_2_Ta_4_O_11_, distortions were observed for PbTa4O11 when it was prepared from Ag2Ta4O11 by a Pb-ion exchange reaction. Similar to the parent structure of Ag_2_Ta_4_O_11_ ($\text{R}\bar{3}\text{c}$), the lower-symmetry PbTa_4_O_11_ (*R3*) exhibits two symmetry-unique layers of edge-shared TaO_7_ pentagonal bipyramids (Ta1 and Ta2), layers of TaO_6_ octahedra (Ta3 and Ta4), and Pb(II) cations (Pb1 and Pb2), as shown in Figure 7 [35]. Each of the isolated TaO_6_ octahedra is surrounded by three monocapped trigonal prismatic PbO_7_ polyhedron that alternate in their orientation, (i.e., [001] for Pb1 and [001] for Pb2) [35]. The PbO_7_ polyhedra are formed by six apical O atoms and one equatorial O atom (Pb1-O7, Pb2-O9) from the adjacent TaO_7_ layers that result in the asymmetric coordination environment [35]. Divalent Pb cations commonly undergo intra-polyhedral distortions due to their high degree of polarizability and their stereoactive electron lone pair, and thereby adopt stable asymmetric anion coordination environments (i.e., PbO_7_) [59,61,66–68]. The stabilization of the Pb(II) cations results in their displacement towards the Ta(V) cations, which in turn electrostatically repulses the Ta atoms (Ta1 and Ta2) from the center of their TaO_7_ polyhedra towards their apical O atoms [35]. Similarly, the Ta atoms in the TaO_6_ octahedra were displaced towards the octahedral faces away from the Pb(II) cations, analogous to the polar distortions reported for Ag_2_Nb_4_O_11_ (R3) [43]. Displacement of the Ta(V) and the Pb(II) cations along the c-axis culminated in alternating expanded and contracted TaO_7_ pentagonal bipyramidal interlayer distances [35].

**Figure 8.**
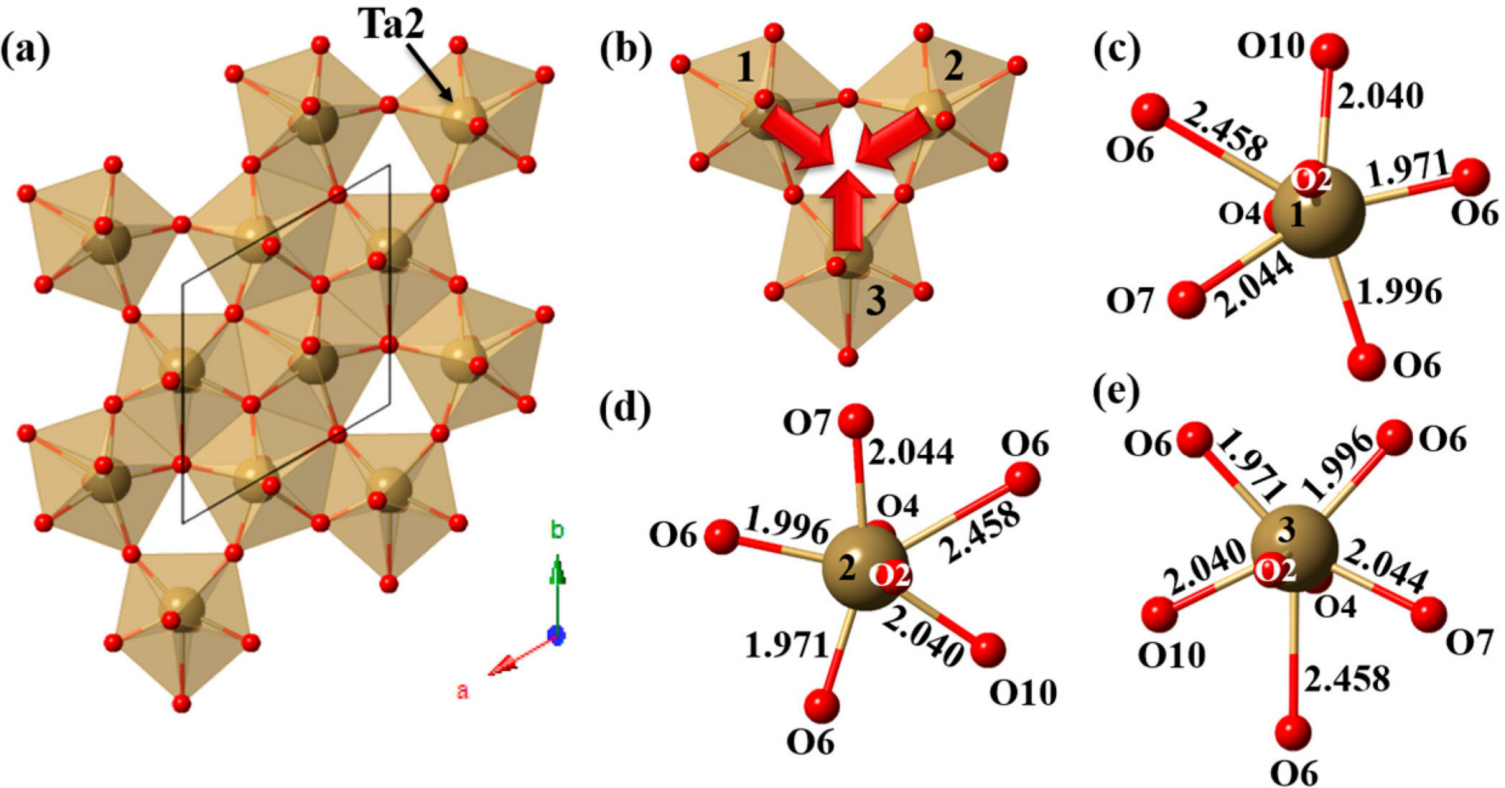
Polyhedral view and local coordination environments of one layer of PbTa_4_O_11_. Shown are (**a**) a polyhedral view of the TaO_7_ layer along the *ab* plane with the unit cell outline in black; (**b**) a smaller segment from the lower right cluster of three labeled Ta2 atoms, with red arrows indicating the atomic displacement; and (**c**–**e**) the local coordination of the labeled Ta2 atoms. Reproduced with permission from [35], published by Elsevier, 2015.

Similar to the distortions along the c-axis between adjacent tantalate layers, electrostatic repulsion effects within the *ab* plane bring about two symmetry unique TaO_7_ pentagonal bipyramid layers. The off-center displacement of the nearest-neighbor Ta atoms (Ta1 and Ta2) towards empty trigonal cavities within the TaO_7_ layers relieves electrostatic strain caused by cation-cation repulsions. This resulted in two symmetry unique Ta atoms, six different Ta-O equatorial bond distances, and increased the TaO_7_-TaO_7_ nearest-neighbor distances, as shown in Figure 8 [35]. In comparison to the centrosymmetric Ag_2_Ta_4_O_11_ structure, the TaO_7_ layers are comprised of one symmetry unique Ta, two different Ta-O equatorial bond distances, and shorter TaO_7_-TaO_7_ nearest-neighbor distances [43]. The symmetry-lowering polar distortions from the centrosymmetric Ag_2_Ta_4_O_11_ $R\bar{3}c$) to the noncentrosymmetric PbTa_4_O_11_ (*R3*) space group are characteristic of SOJT distortions that are typically observed for structures with high valent d^0^ cations (i.e., Ta(V)) and cations containing filled valence s shells (i.e., Pb(II)). The SOJT distortion reduces the crystal symmetry and is the driving force for stabilizing ionic shifts [59,61,66–68].

### 3.3. Niobates/Tantalates Solid Solution

The polymorphism of the (Na_1−*x*_Ag*_x_*)_2_Nb_4_O_11_ solid solutions have been investigated as a function of chemical composition and temperature by the Woodward and West groups, which revealed a complex system with interesting structural transformations [69,70]. The (Na_1−*x*_Ag*_x_*)_2_Nb_4_O_11_ structures were characterized using Rietveld refinements of variable temperature powder X-ray and neutron diffraction data, differential scanning calorimetry, and lattice parameter trends. The West group reported a monoclinic (*C*2/*c*) to rhombohedral ($R\bar{3}c$) phase transformation upon heating compositions with *x* ≤ 0.25, and a monoclinic *C*2/*c* (*x* = 0.25) to rhombohedral $R\bar{3}c$ phase transformation upon cooling below ≈107 °C. Further, the rhombohedral and monoclinic phases coexist in a two-phase region at the phase boundary for compositions of 0.65 ≤ *x* ≤ 0.8. The origin of the rhombohedral $R\bar{3}c$ to monoclinic *C*2/*c* phase transition is related to distortions within the equatorial plane of the NbO_7_ pentagonal bipyramids, as described below [69,70].

An interesting series of ferroelectric-paraelectric phase transformations was observed using variable temperature powder X-ray diffraction and lattice refinements. The transformation from *R3* to *R3c* to $R\bar{3}c$ occurred upon heating to higher temperatures for compositions where *x* ≥ 0.85. For example, upon cooling the $\text{R}\bar{3}\text{c}$ phase (*x* = 0.875) it transforms to *R3c* at ≈87 °C and to *R3* at ≈−63 °C. The series of ferroelectric-paraelectric phase transformations are related to the displacement of the Nb atoms in the NbO_7_ pentagonal bipyramids towards the apical O atoms, as well as displacement of Nb atoms in NbO_6_ and Na/Ag atoms towards their octahedral faces. A triclinic $P\bar{1}$ crystal structure was observed in the compositional range of 0.25 < *x* < 0.60 in the temperature range −273 °C to 3 °C [70]. Heating the low-temperature $P\bar{1}$ crystal structure resulted in the structural transformation to the monoclinic P2_1_/c at temperatures up to room temperature. At temperatures above room temperature the monoclinic P2_1_/c crystal structure transforms to the rhombohedral $R\bar{3}c$ crystal structure [70]. The triclinic distortions from the rhombohedral structure are the result of a combination of off-center displacement of the Nb atoms in the NbO_7_ and NbO_6_ polyhedra, as well as movement of the equatorial O atoms in the NbO_7_ pentagonal bipyramid layers out of the equatorial plane [70].

The Na_2−*x*_Cu*_x_*Ta_4_O_11_ solid solutions were also investigated by the Maggard group with compositional limits between 0 ≤ *x* ≤ 0.78 at a reaction temperature of 950 °C [71]. The solid solutions did not display any phase transformation; however, Wyckoff site substitution of Na cations and Cu cations in the $R\bar{3}c$ crystal structure of the solid solution were observed together with a significant decrease in bandgap size from ≈4.0 eV to ≈2.65 eV [71]. The origin of the differential site occupancy of the Ag and Cu cations is driven by the preferred coordination number of the Na/Cu cation to the surrounding O atoms [71]. The linearly coordinated Cu cation and 7-coordinate Na cation are located at the 18d and 12c Wyckoff crystallographic sites, respectively. By comparison, the use of nanoparticle reactants and lower reaction temperatures of ≈625 °C to ≈700 °C were found necessary to obtain the Cu-richest composition, i.e., Cu_2_Ta_4_O_11_, in high crystalline purity[65]. This composition was found to undergo a transformation from a monoclinic structure (*Cc*) to a rhombohedral ($R\bar{3}c$) crystal structure upon heating, i.e., from *α*-Cu_2_Ta_4_O_11_ to β-Cu_2_Ta_4_O_11_, as described above [64].

## 4. Photocatalysis

### 4.1. Background

Among renewable energy sources, solar energy is the largest, exploitable resource that could meet current and future human energy demand, with an estimation that ≈0.015% of solar energy reaching the earth would be enough to support human civilization [72–75]. The capture and conversion of solar energy to drive photocatalytic hydrogen production would provide a clean alternative energy solution that can be used to generate useful chemical fuels Honda and Fujishima were the first to demonstrate the decomposition of H_2_O into H_2_ and O_2_ using TiO_2_ as an *n*-type photocatalyst [76]. Since this groundbreaking work, semiconductor-based photocatalytic water splitting to produce hydrogen has been considered one of the most important approaches to solving the world energy crisis [77]. Semiconductors have also been used in practical application for the photocatalytic degradation of organic pollutants in water [78–81]. The photoelectrolysis of water using semiconductors as both light absorber and energy converter (i.e., catalyst) to store the solar energy in the simplest bond, H_2_, has been described as the “Holy Grail” of solar energy conversion and storage [74,82–84].

**Figure 9.**
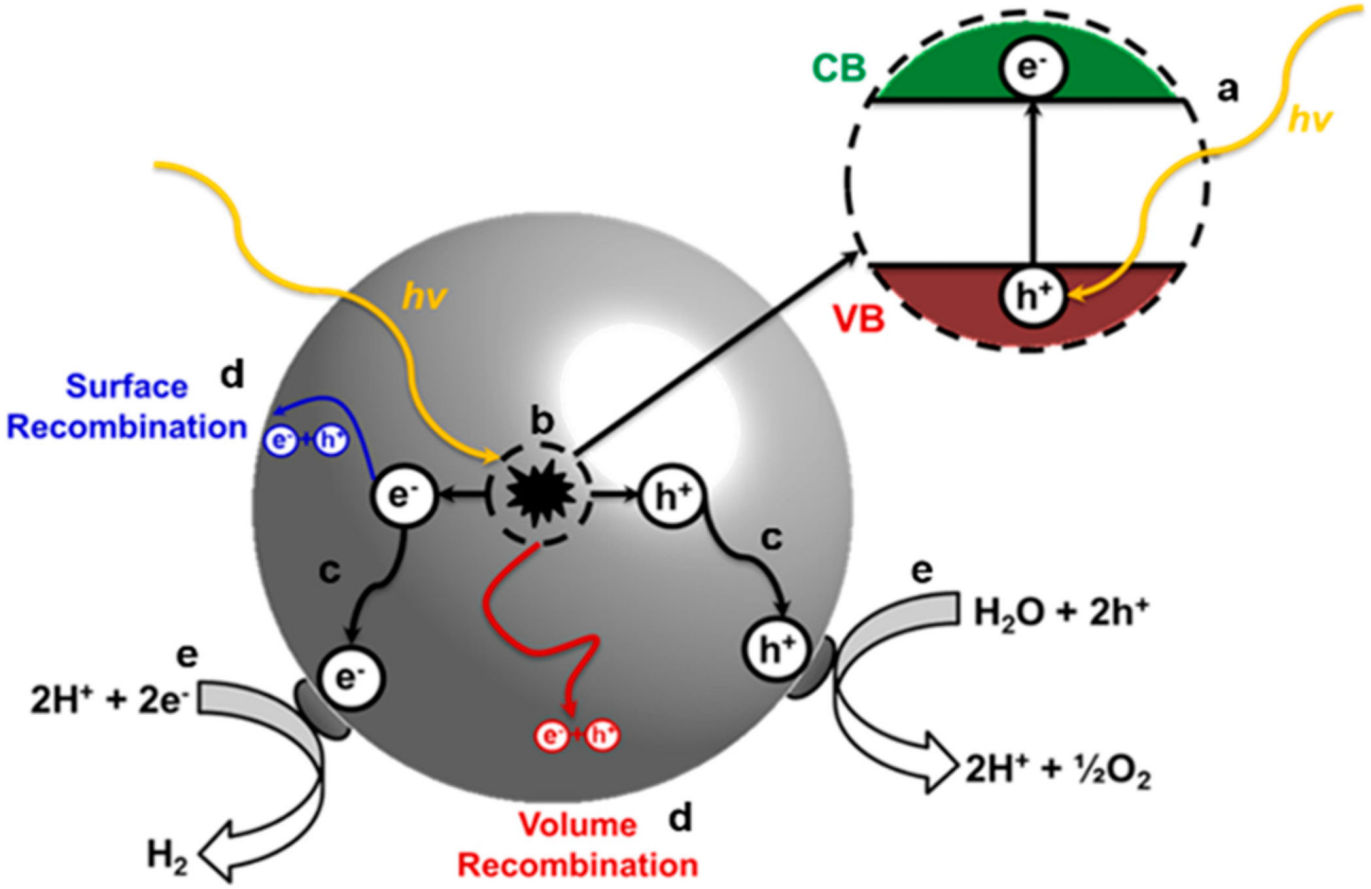
An illustration of a particulate photocatalyst, showing the processes of (**a**) light absorption; (**b**) charge separation; (**c**) charge migration; (**d**) recombination; and (**e**) redox reaction.

**Figure 10.**
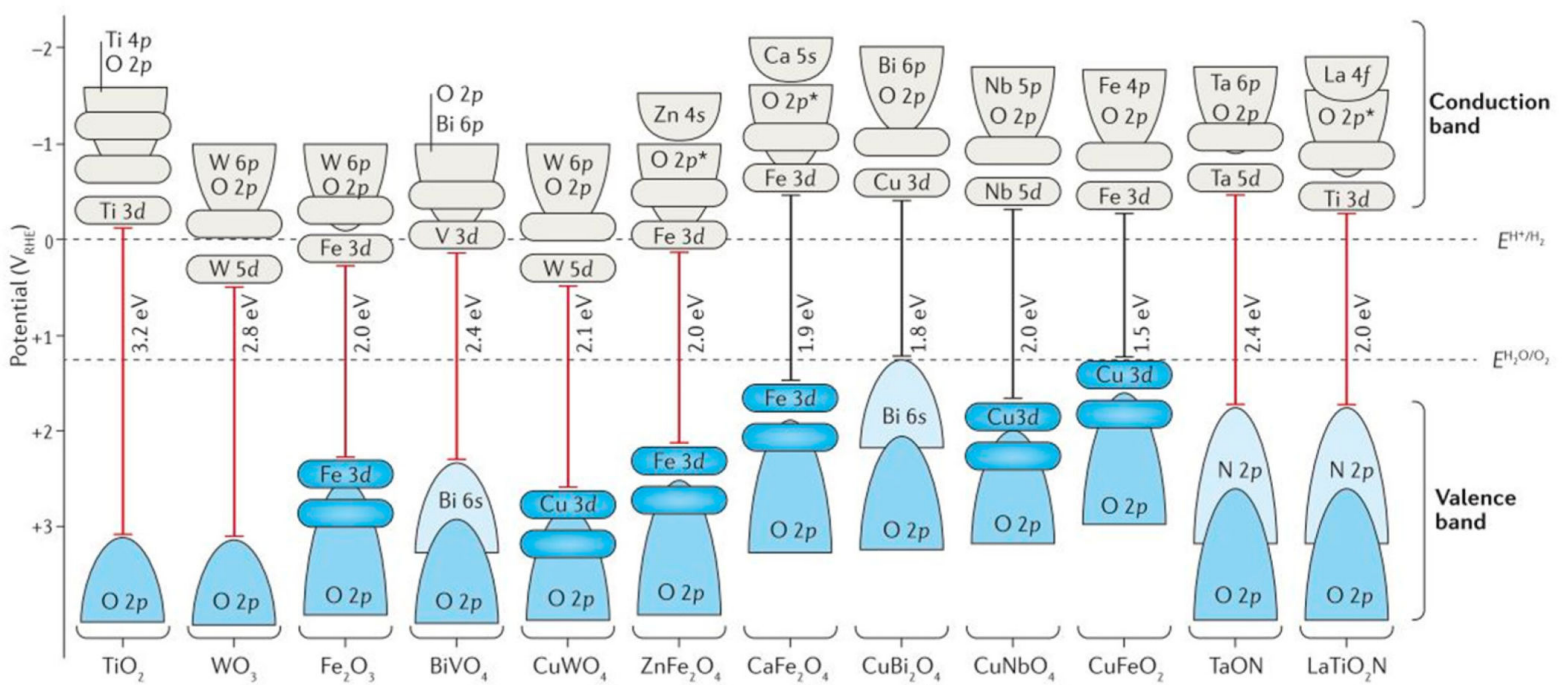
The bandgap energies (red for *n*-type, black for *p*-type) are shown with respect to the reversible hydrogen electrode and the water oxidation redox couple (assuming Nernstian behavior for the band-edge energies with respect to the electrolyte pH). The contribution of the valence and conduction band orbitals are outlined in blue and grey, respectively. Reproduced with permission from [101], published by Macmillian Publishers Limited, Part of Springer Nature, 2016.

Photocatalysis for water splitting can be divided into four mechanistic steps: (a) absorption of photons to excite electrons from the valence band to the conduction band; (b) charge separation electrons and holes; (c) diffusion of the photoexcited electrons and holes to the surfaces as driven by the space-charge layer, and (d) surface chemical reactions between the charge carriers and the adsorbates [85–89]. The illustration in Figure 9 shows a photocatalyst particulate and the four basic steps, where (d) also includes the competing volume and surface recombination processes. An efficient photocatalyst must have band gap energies capable of the absorption of visible light energies and possess energetic band positions suitable for driving water reduction and oxidation reactions (i.e., E_g_ ≥ 1.23 eV) [90]. The valence and conduction band energies of several semiconductors are plotted in Figure 10, and shown relative to the water redox couples for water splitting. Additionally, the photocatalyst must be stable in aqueous solution under solar irradiation. The use of co-catalysts on semiconductors has proven to be an effective approach to promote charge separation, transfer, and to suppress recombination and back-reactions during photocatalysis [91]. The electronic structure of the semiconductor also determines the functionality and efficiency of a photocatalyst. This is not limited to the space charge width alone, which is composed of the accumulation layer, depletion layers, and inversion layer. The carrier diffusion lengths, and charge carrier mobility are also critical to the material’s photocatalytic performance [85,92–95]. In addition, selecting a suitable doping strategy is essential to enhancing photocatalytic properties [96]. Common challenges for achieving water splitting with heterogeneous photocatalysts include rapid recombination of electrons/holes [97], hole/electron collection at particle surfaces [98], and improvement of the photocatalytic rates [99,100].

Evaluation of photocatalysts under experimental conditions suitable for water splitting is dependent upon the following: the evolution of O_2_ and H_2_ gases, stability over time, as a function of wavelength (i.e., ultraviolet or visible light), and overall efficiency of the system [101–105]. Depending upon the overall configuration of the photocatalytic measurements, (i.e., whether in the form of suspended particles or as a polycrystalline film) various parameters are important to assess solar-to-fuel energy conversion, including the photocurrent density, turnover frequency, overall quantum yield,

(1) $$\text{Overall Quantum Yield}=\frac{rate\;of\;reaction}{rate\;of\;absorption\;of\;radiation}=\frac{N_{mol}}{N_{ph}}$$

and the incident photon-to-current-efficiency (IPCE). The photoelectrochemical properties of polycrystalline films of the Cu(I) tantalates of this structural family (e.g., Cu_5_Ta_11_O_30_) have been recently reviewed [85], and this current review will focus on their photocatalytic activity as suspended powders in aqueous solutions.

For investigations where solid photocatalysts particles are suspended in an aqueous solution and irradiated, the rate of hydrogen and/or oxygen production and the quantum yield are commonly reported. A turnover number can be calculated from the moles of evolved H_2_ and the number of surface active sites of the photocatalyst. The latter can be approximated from surface area measurements and a knowledge of the crystalline structure, and this is typically reported in units of μmol·h^−1^·g^−1^. The overall quantum yield, listed in Equation (1), is defined as the number of molecules produced relative to the total number of incident photons in a reaction vessel [83]. The number of molecules N_mol_ undergoing an event (conversion of reactants or formation of products) relative to the number of quanta (photons) N_ph_ absorbed by the reactants or photocatalyst is determined based on the cm^3^ per seconds (cm^3^/s) of the reactions.

Heterogeneous photocatalysts for water reduction or oxidation are common among many metal-oxide systems, which include binary oxides (i.e., TiO_2_, Cu_2_O, Fe_2_O_3_, WO_3_), oxynitride systems (i.e., TaO_x_N_y_ BaTaO_2_N, LaTiO_2_N), layered oxides (K_4_Nb_6_O_17_, Na_2_W_4_O_13_, RbLaNb_2_O_7_), and mixed-metal oxides (BiVO_4_, CuFeO_2_, CuWO_4_) [106–113]. In addition perovskite-based photocatalysts have been extensively studied and various strategies have been employed for enhancing photocatalytic performance [90,114,115]. The use of mixed-metal compositions is critical to lowering the bandgap sizes of simpler metal oxides into the visible region [116]. Layered oxide systems are also of importance in photocatalytic systems due to their unique interlayer spaces in which reduction sites are separated from oxidation sites, yielding among the highest rates for hydrogen and oxygen production from solar energy [117–119]. However, the incorporation of secondary transition metals into the interlayer sites, (i.e., to lower their bandgap sizes into the visible range) is typically not a stable configuration in aqueous solutions where the layers can delaminate. Thus, the family of layered structures based pentagonal bipyramid layers serve as an alternative for achieving efficient water splitting activity in aqueous solutions. In addition, several members within the system are comprised of early and late transitions metals (i.e., Cu^1+^/Ta^5+^, Ag^1+^/Nb^5+^, Ag^1+^/Ta^5+^) which possess lower visible-light band gaps that are suitable for use with the solar spectrum and capable of driving the water splitting reactions.

The Maggard group has extensively investigated the visible and ultraviolet photocatalytic activity of members from the ${{\text{A}}^{m+}}_{\left((n+1)/m\right)}{\text{B}}_{\left(3n+1\right)}{\text{O}}_{\left(8n+3\right)}$ (e.g., A = Na, Ag, Cu, Pb, Bi; B = Nb, Ta) family of structures as both suspended particles and polycrystalline films for water splitting [92,116,120,121]. Improved charge separation and photocatalytic/photoelectrochemical redox reactions have been proposed to be enhanced in these structures due to the preferential anisotropic diffusion of charge carriers along the BO_7_ pentagonal bipyramid layers. The anisotropic charge diffusion is a result of the delocalization of the Nb 4d and the Ta 5d lowest-energy unoccupied crystal orbitals across the BO_7_ pentagonal bipyramid layers, as confirmed by electronic structure calculations which show the largest band dispersion in these crystallographic directions [35,47,64,65,71,122–128].

### 4.2. Photocatalysts with Single MO_7_ Layers

**Figure 11.**
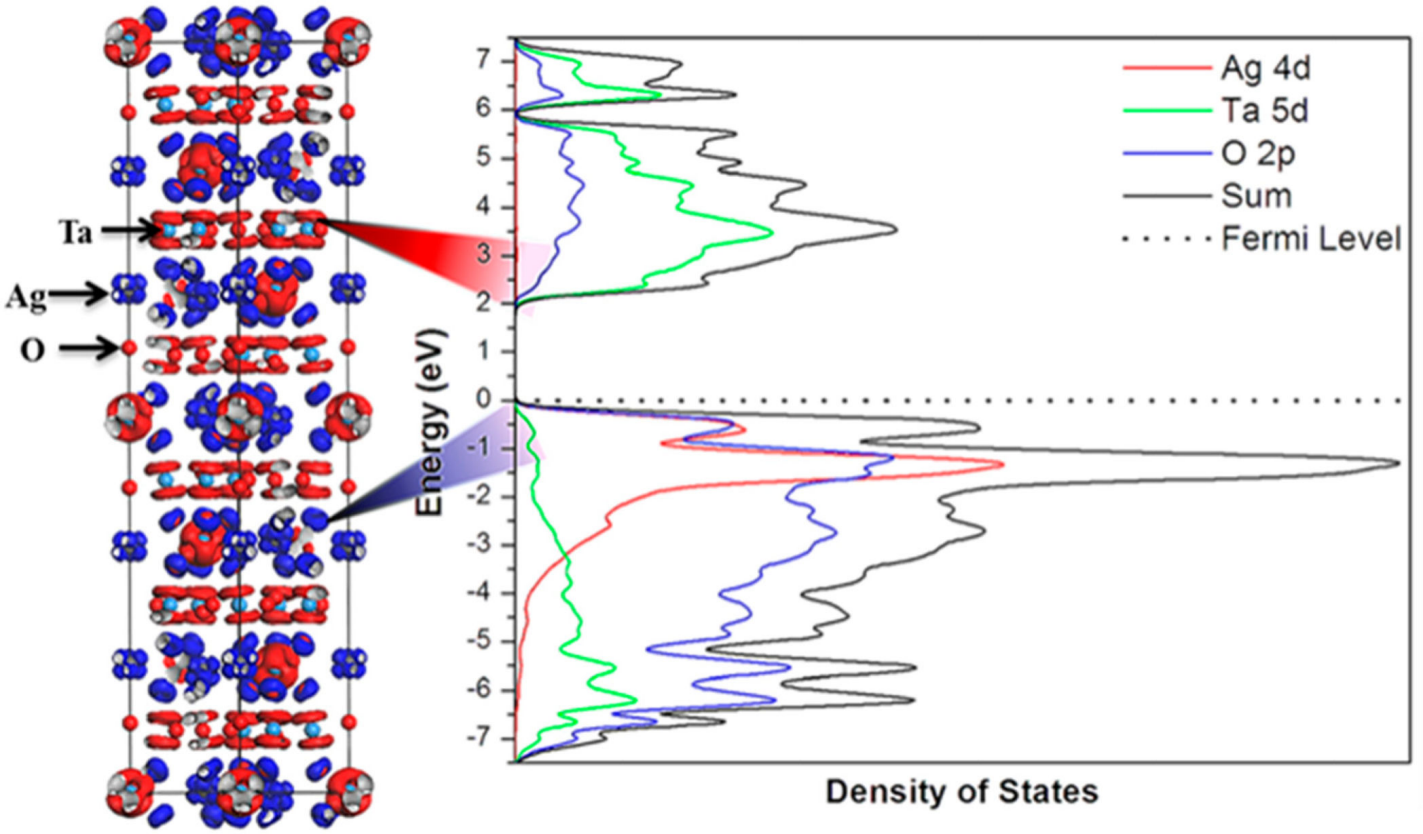
The densities-of-states (DOS; **right**) and electron density plots (**left**) of Ag_2_Ta_4_O_11_. The individual atomic contributions are projected out in the DOS, and the electron density at the top of the valence band and bottom of the conduction band are shaded red and blue, respectively. Reprinted with permission from [126]. Copyright 2013 American Chemical Society.

The first system investigated in this family of structures for photocatalytic activity was the natrotantite compound, Na_2_Ta_4_O_11_, which showed activity for hydrogen production under ultraviolet-visible light of ≈13.4 to ≈34 μmol H_2_·h^−1^·g^−1^ [127]. Owing to its large bandgap size of ≈4.3 eV, its activity was limited to ultraviolet light wavelengths. The variation in H_2_ gas rates were dependent upon the synthetic conditions and the particle sizes and surface areas of the polycrystalline powders [127]. The particle sizes varied with a wide distribution from ≈100 nm to >≈1000 nm among all of the products that were investigated by scanning electron microscopy, with low total surface areas from ≈0.8 to ≈1.0 m^2^/g. Notably, the highest photocatalytic rates for hydrogen production were found for crystallites with highly faceted and nanoterraced surfaces. These rates were stable over the course of several hours during the photocatalytic reactions [127]. Similarly, the Ag(I) analogues, i.e., Ag_2_M_4_O_11_ (A = Ta, Nb), have been investigated as photocatalysts for water splitting and organic dye degradation [124,126,129]. The Ag_2_Nb_4_O_11_ composition has a smaller band gap of ≈3.1 eV, while for Ag_2_Ta_4_O_11_ it is ≈3.9 eV. As a suspended powder, the Ag_2_Ta_4_O_11_ compounds shows photocatalytic activity for hydrogen production of ≈23 μmol H_2_·h^−1^·g^−1^. This is similar in rate to that found for Na_2_Ta_4_O_11_. However, the activity for oxygen production was significantly higher ≈165 μmol O_2_·h^−1^·g^−1^, and higher compared to that for Na_2_Ta_4_O_11_ of ≈110 μmol O_2_·h^−1^·g^−1^. Both compounds crystallize in the same centrosymmetric rhombohedral space group, which suggests the higher activity for oxygen production arises from the new Ag-based valence band in Ag_2_Ta_4_O_11_, shown in Figure 11. By comparison, the Ag_2_Nb_4_O_11_ compound is ferroelectric and crystallizes in the polar R3c space group at room temperature. While the Ag_2_Nb_4_O_11_ compound has been found active for photocatalytic dye degradation [129], there are currently no reports of its photocatalytic activity for water reduction or oxidation.

**Figure 12.**
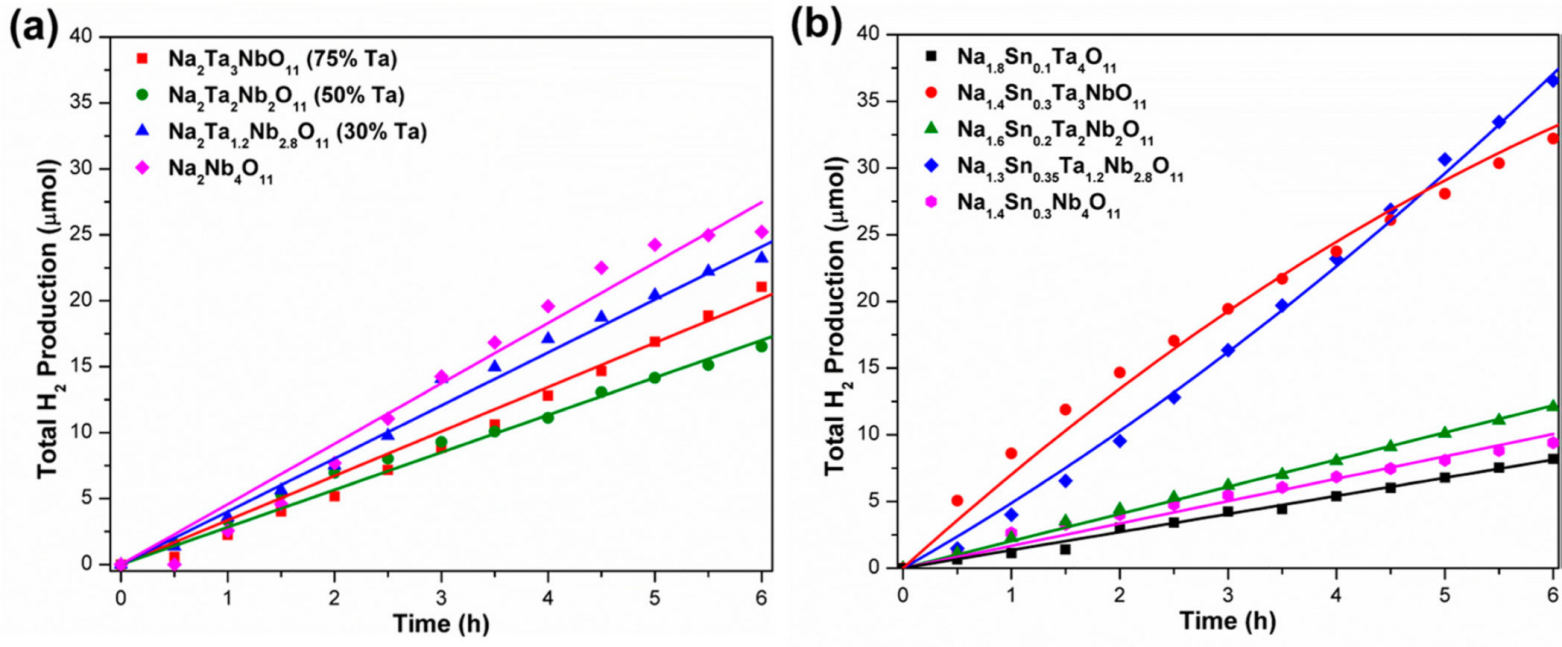
Photocatalytic hydrogen production (μmol H_2_) versus time (h) for (**a**) Na_2_Ta_4_−*y*Nb_*y*_O_11_ and (**b**) Na_2−2*x*_Sn_*x*_Ta_4−*y*_Nb_*y*_O_11_ under ultraviolet-visible (λ > 230 nm) irradiation. Reprinted with permission from [125]. Copyright 2016 American Chemical Society.

More recently, the solid solutions of Na_2_Ta_4−*y*_Nb*_y_*O_11_ (1 ≤ *y* ≤ 4) were investigated for hydrogen production [125]. All solid solution compositions investigated were photocatalytically active for the generation of hydrogen and exhibited higher rates for hydrogen production in comparison to Na_2_Ta_4_O_11_ and Ag_2_Ta_4_O_11_ [125], as shown in Figure 12. Mott-Schottky measurements were evaluated in order to determine the positions of the conduction and valence band edges for selected samples in the Na_2_Ta_4−*y*_Nb*_y_*O_11_ solid solution. The conduction band energies followed a trend toward more positive potentials and resulted in a red-shift in the absorption edge owing to the increase in lower energy Nb 4d orbitals [125]. The bandgap size decreased across the solid solution series from ≈4.3 eV for Na_2_Ta_4_O_11_ to ≈3.6 eV for Na_2_Nb_4_O_11_. The highest photocatalytic rate for hydrogen production was exhibited by Na_2_Nb_4_O_11_, which generated 84 μmol H_2_·h^−1^·g^−1^ under ultraviolet-visible light (λ > 230 nm) [125]. At an *x ≈*2.7 to 3.0, (i.e., ≈67% to ≈70% Nb content) the structural transformation from the rhombohedral to monoclinic space groups occurs, as described above, both of which are centrosymmetric. However, the general trends in bandgap size and photocatalytic activity do not show any discontinuities, but rather, both gradually shift and result in lower band energies and higher photocatalytic activities with increasing Nb content [125]. This suggests the higher activities are the result of increased light absorption, and are not significantly impacted by this specific structural transformation. The further substitution of Sn (II) cations into the structure was partially successful, resulting in a Sn (II) content that varied between ≈11% to ≈21%. Significant red-shifting of the bandgap size occurred down to ≈2.3 eV into the visible light energies, as well as higher photocatalytic activities for hydrogen formation of up to ≈124 μmol H_2_·h^−1^·g^−1^ under ultraviolet and visible-light energies, as shown in Figure 12 [125]. Further, the conduction and valence band energies were both found to maintain suitable positions for driving both the reduction and oxidation of water. However, further research is necessary to elucidate any structural changes and the crystallographic location of the Sn(II) cations, as well as the preparation of a fully Sn(II) exchanged composition (i.e., SnTa_4_O_11_ in analogy with PbTa_4_O_11_).

Among the divalent cations of this family, CaTa_4_O_11_ and the related solid solution Ca_1−*x*_Sr*_x_*Ta_4_O_11_, 0 ≤ *x* ≤ 0.3, has been investigated for suspended particle photocatalysis [130]. The bandgap size across this series ranged from ≈4.4 eV to ≈4.2 eV with increasing Sr content, photocatalytic activities were limited to ultraviolet irradiation. The rate of hydrogen production varied, from ≈502 μmol/h with a NiO cocatalysts to ≈20–30 μmol/h without a cocatalyst [130]. Photocatalytic activity was dependent upon the preparation of the sample, co-catalyst, and loading methods of the cocatalyst. Generally, no apparent trends were observed in the rates for increasing amounts of Sr substitution into the compound [130]. The samples were prepared using either a polymerized complex method or impregnation method [130]. For pure CaTa_4_O_11_, rates for hydrogen production were found to be ≈403 μmol/h of H_2_ when loaded with 0.5 wt % of NiO co-catalyst in comparison to a rate ≈62 μmol/h of H_2_ when RuO_2_ was used as the co-catalyst. The solid solution with the highest activity also had the highest Sr content, (i.e., Ca_0.7_Sr_0.3_Ta_4_O_11_) yielded ≈575 μmol/h of H_2_ in the presence of NiO co-catalysts on the surface of the photocatalyst and ≈16 μmol/h of H_2_ without the addition of a co-catalyst [130]. The cited reason for the higher activity was the relaxation of the structural distortion when substituting the larger Sr cation into the structure. The rates for oxygen production followed generally similar trends, which peaked varied between ≈11 μmol/h to ≈300 μmol/h. Thus, relatively high rates of both hydrogen and oxygen production are found, and a high quantum yield of ≈5.8% was measured under 254 nm irradiation for total water splitting for Ca_0.7_Sr_0.3_Ta_4_O_11_ with 0.5 wt % of NiO as a surface co-catalyst.

**Figure 13.**
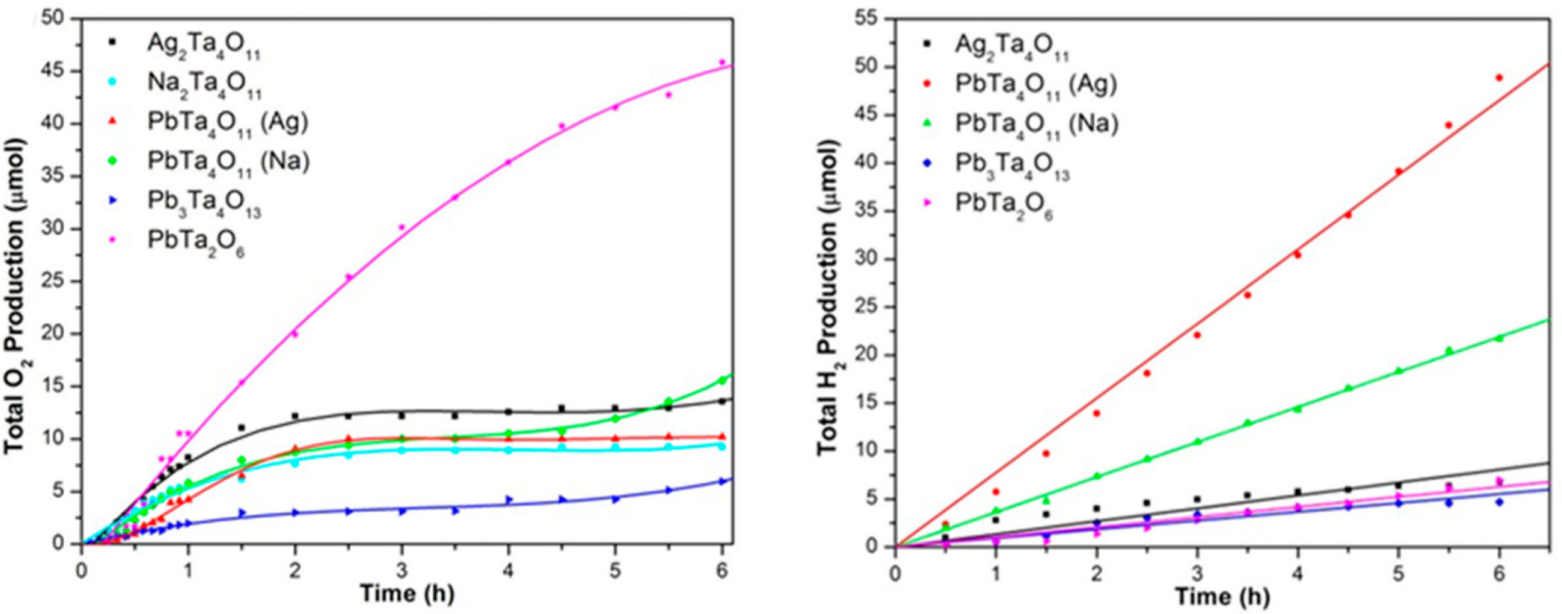
Photocatalytic oxygen production (μmol O_2_; **left**) and hydrogen (μmol H_2_; **right**) versus time (h) for the M_2_Ta_4_O_11_, (M = Ag, Ta), and Pb_2_Ta_4_O_11_, Pb_3_Ta_4_O_13_, and PbTa_2_O_6_ phases under ultraviolet-visible (λ > 230 nm) irradiation. Adapted with permission from [126]. Copyright 2013 American Chemical Society.

The photocatalysis of PbTa_4_O_11_ for hydrogen and oxygen production was investigated under ultraviolet-visible light irradiation, shown in Figure 13. The PbTa_4_O_11_ phase was prepared in high purity by Pb(II) exchange of either Ag_2_Ta_4_O_11_ or the Na_2_Ta_4_O_11_ compounds and shows a symmetry-lowering to a polar space group [35,126]. The PbTa_4_O_11_ phase exhibits a relatively high rate for hydrogen production of ≈175 μmol H_2_·g^−1^·h^−1^ when prepared from the Ag_2_Ta_4_O_11_ precursor, and a lower rate of ≈72 μmol H_2_·g^−1^·h^−1^ when prepared from Na_2_Ta_4_O_11_ [126]. These differences were partially attributed to the higher amounts of Pb^2+^ cations at the surfaces of PbTa_4_O_11_ prepared from Ag_2_Ta_4_O_11_. By comparison, both Na_2_Ta_4_O_11_ and Ag_2_Ta_4_O_11_ gave lower rates of ≈10 to ≈35 μmol H_2_·g^−1^·h^−1^ [126]. However, a further comparison of these rates with those for Ca_1−x_Sr_x_Ta_4_O_11_ is not possible owing to the different testing conditions and surface co-catalysts. Shown in Figure 13 (left), the photocatalytic rates for O_2_ production were calculated from the initial rates over 1 h for the PbTa_4_O_11_ phase and its precursor, Ag_2_Ta_4_O_11_. The rates of O_2_ production decreased over time as Ag(s) is deposited onto the particles’ surfaces owing to its reduction as a sacrificial reagent. The oxygen production of the PbTa_4_O_11_ phase was found to be ≈84 μmol O_2_·g^−1^·h^−1^, while its precursor Ag_2_Ta_4_O_11_ exhibited a higher rate of ≈165 μmol O_2_·g^−1^·h^−1^[126]. The PbTa_4_O_11_ phase with a 1 wt % platinum co-catalyst exhibited an overall water splitting rate, i.e., for both hydrogen and oxygen production, at a rate of ≈34 μmol gas g^−1^·h^−1^ in deionized water under ultraviolet-visible irradiation [126]. The bandgap size of Ag_2_Ta_4_O_11_ and PbTa_4_O_11_ are relatively the same (≈3.8 eV). The symmetry-lowering structural distortions and resulting electronic distortions in the PbTa_4_O_11_ phase are associated with its relatively higher photocatalytic rates for hydrogen generation compared to other undistorted single-layered structures (i.e., Na_2_Ta_4_O_11_ and Ag_2_Ta_4_O_11_). Out-of-center distortions of d^0^ transition metal cations have been reported to result in the generation of internal electric fields that can aid in electron/hole charge separation in polar noncentrosymmetric metal oxides, similar to that observed in the PbTa_4_O_11_ phase [61,131,132]. The improved electron/hole charge separation due to the internal electric fields can aid in mitigating the effects of recombination and back-reaction, and act to increase photocatalytic hydrogen generation [35].

### 4.3. Double MO_7_ Layered Photocatalysts

The double-layered BiTa_7_O_19_ phase has exhibited hydrogen production under ultraviolet-visible light irradiation in 20% methanol solution as a suspended particle photocatalyst with a rate of ≈194 μmol H_2_·g^−1^·h^−1^. similar to PbTa_4_O_11_ [126]. Analogous to the PbTa_4_O_11_ phase, the photocatalytic rates for O_2_ production were calculated from the initial 1 h rate for BiTa_7_O_19_. The BiTa_7_O_19_ phase was found to exhibit a rate of ≈140 μmol O_2_·g^−1^·h^−1^. The BiTa_7_O_19_ phase with a 1 wt % platinum co-catalyst exhibited total water splitting in deionized water under ultraviolet-visible irradiation with a rate of ≈34 μmol gas g^−1^·h^−1^ [126]. Anisotropic migration of charge carriers along the TaO_7_ pentagonal bipyramid double layers in BiTa_7_O_19_ may aid in charge separation and facilitate its higher suspended particle photocatalytic activity. The suspended particle photocatalytic gas generation rates of double layered metal-oxides have been reported to be some of the highest, such as layered perovskites, and may account for its reported higher activity in comparison to the single-layered A_2_Ta_4_O_11_ (A = Na, Ag) phases [24,113,126,133].

The suspended particle photocatalytic rates of the related Cu (I)-tantalates and -niobates have not been as intensely investigated currently. In a recent example, Cu_3_Ta_7_O_19_ was investigated for suspended particle photocatalysis where H_2_ was produced at the relatively rate of 0.1 μmol H_2_·h−1·g− [119]. Rather, these materials have been investigated in the form of polycrystalline films as p-type semiconductors, and which show significant cathodic photocurrents in the range of −0.5 to −3.0 mA/cm^2^, but these efforts have been covered more extensively in a recent review [85].

## 5. Conclusions

The crystallography and photocatalytic properties of a polysomatic family of ternary mixed-metal oxides, ${{\text{A}}^{m+}}_{\left((n+1)/m\right)}{\text{B}}_{\left(3n+1\right)}{\text{O}}_{\left(8n+3\right)}$ (e.g., A = Ag, Bi, Ca, Cu, Ce, Dy, Eu, Gd K, La, Nd, Pb, Pr, Sr, Y; B = Nb, Ta); *m* = 1–3; *n* = 1, 1.5, 2), which feature the layers of pentagonal bipyramids contained in the *α*-U_3_O_8_ structure type (with mixed six- and seven-coordination sites) are reviewed. These compounds highlight either single, double, triple, or mixed layers of edge-sharing BO_7_ pentagonal bipyramid layers alternating with isolated octahedral layers, as well as A-site cations with 2-, 6-, 7-, or 8-coordination depending on their oxidation state. Some of the members exhibit symmetry lowering phase transitions as a function of chemical composition, temperature, and reaction conditions. These symmetry lowering structure distortions, and the resulting electronic distortions have important consequences for photocatalytic applications. As illustrated by the PbTa_4_O_11_ phase which shows an out-of-plane distortion of the d^0^ transition metal cation, an internal electric dipole can facilitate the electron/hole charge separation, thus mitigating the effects of recombination and back-reaction. It was also found that the higher charge of the cation A (for example in BiTa_7_O_19_) can help charge separation due to the anisotropic migration of charge carriers along the BO_7_ pentagonal bipyramid double layers, giving rise to higher photocatalytic rate for hydrogen generation.

## Supplementary Material

Supp1
