# Supplementary material for: Polymorphism and Structural Distortions of Mixed-Metal Oxide Photocatalysts Constructed with α-U3O8 Types of Layers
Source: Crystals (Basel). Author manuscript; Available in PMC 2024 Oct 22. (PMC11494910; doi:10.3390/cryst7050145)
Supplement: Supp1 [file NIHMS1530787-supplement-Supp1.pdf]

Supplementary Materials

Polymorphism and Structural Distortions of Ternary Mixed-Metal Oxide Photocatalysts Constructed with α-U_3O_8 Types of Layers

Nacole King[1*], Jonathan Boltersdorf [2], Paul Maggard[3], Winnie Wong-Ng[1]

Table S1. A table of selected members comprised of single pentagonal bipyramid layers alternating with octahedral layers. The space group, unit cell parameters, and interatomic distances are listed for symmetry unique MO_7 pentagonal bipyramids (M = Nb, Ta) are included.

Layer	Chemical Formula	Space Group	*a* (Å)	*b* (Å)	*c* (Å)	Interatomic distances (Å)	Ref.
**n=1, single**	$Ag_2Nb_4O_{11}$	$R\bar{3}c$	6.2301(1)	6.2301(1)	37.0163(7)	NbO_7: Nb-O 1.9730(11) x2<br>Nb-O 2.0032(10) x2<br>Nb-O 2.0216(21) x2<br>Nb-O 2.4207(21)	[43]
		R3C	6.2080(1)	6.2080(1)	37.0135(7)	NbO_7: Nb-O 1.945(7)<br>Nb-O 1.981(6)<br>Nb-O 2.001(8)<br>Nb-O 2.010(7)<br>Nb-O 2.013(9)<br>Nb-O 2.043(6)<br>Nb-O 2.391(2)	[43]
		R3	6.20524(9)	6.20524(9)	37.0175(6)	NbO_7: Nb1-O 1.878(15)<br>Nb1-O 2.006(9)<br>Nb1-O 2.024(15)<br>Nb1-O 2.026(15)<br>Nb1-O 2.039(10)<br>Nb1-O 2.064(16)<br>Nb1-O 2.304(9)<br>Nb2-O 1.904(16)<br>Nb2-O 1.982(9)<br>Nb2-O 1.984(16)<br>Nb2-O 1.998(15)<br>Nb2-O 2.036(10)<br>Nb2-O 2.083(14)<br>Nb2-O 2.490(9)	[43]
	$Ag_2Ta_4O_{11}$	$R\bar{3}c$	6.2075(1)	6.2075(1)	36.8605(6)	TaO_7: Ta-O 1.9736(10) x2<br>Ta-O 2.0026(9) x2<br>Ta-O 2.0133(5) x2<br>Ta-O 2.3983(18) x2	[43]

Layer	Chemical Formula	Space Group	*a* (Å)	*b* (Å)	*c* (Å)	Interatomic distances (Å)	Ref.
**n=1, single**	$Cu_2Ta_4O_{11}$	$R\bar{3}c$	6.2190(2)	6.2190(2)	37.107(1)	TaO_7: Ta-O 1.9972(8)<br>Ta-O 1.9973(8)<br>Ta-O 1.994(2)<br>Ta-O 1.994(3)<br>Ta-O 2.021(2)x2<br>Ta-O 2.44(1)	[66]
		Cc	10.734 (1)	10.734 (1)	10.734 (1)	TaO_7: Ta-O 1.92(2) x2<br>Ta-O 1.98(1) x2<br>Ta-O 2.04(1) x2<br>Ta-O 2.46(3)	[65]
	$K_2Ta_4O_{11}$	$R\bar{3}c$	6.280(3)	6.280(3)	36.8769(10)		[41]
	$Na_2Nb_4O_{11}$	$R\bar{3}c$	6.2279(1)	6.2279(1)	36.7264(7)	NbO_7: Nb-O 1.9638(10) x2<br>Nb-O 2.0073(10) x2<br>Nb-O 2.0216(6) x2<br>Nb-O 2.4136(21)	[42]
		C2/c	10.84	6.162	12.75	NbO_7: Nb-O 1.96(2)<br>Nb-O 1.96(3)<br>Nb-O 1.99(2)<br>Nb-O 2.01(1)<br>Nb-O 2.02(3)<br>Nb-O 2.04(3)<br>Nb-O 2.38(2)	[38]
	$Na_2Ta_4O_{11}$	$R\bar{3}c$	6.198(3)	6.198(3)	36.56(2)	TaO_7: Ta-O 1.953(14) x2<br>Ta-O 2.009(7) x2<br>Ta-O 2.010(6) x2<br>Ta-O 2.379(23)	[64]
	$PbTa_4O_{11}$	*R3*	6.23700 (2)	6.23700 (2)	36.8613 (1)	TaO_7: Ta1-O 1.944(8)<br>Ta1-O 1.971(8)<br>Ta1-O 2.013(7)<br>Ta1-O 2.014(7)<br>Ta1-O 2.032(11)<br>Ta1-O 2.0389(11)<br>Ta1-O 2.379(4)<br>Ta2-O 2.3983(18)<br>Ta2-O 1.9736(10)<br>Ta2-O 2.0026(9)<br>Ta2-O 2.0133(5)<br>Ta2-O 2.3983(18)<br>Ta2-O 1.9736(10)<br>Ta2-O 2.0026(9)	[35]
	$SrTa_4O_{11}$	$P6_322$	6.2543(1)	6.2543(1)	12.3320(3)	TaO_7: Ta-O 1.955(1) x2<br>Ta-O 1.980(1) x2<br>Ta-O 2.0598(6) x2<br>Ta-O 2.478(2)	[39]

Table S2. The general trends for extended structure increase in the "A" site coordination number is directly correlated to decreasing c lattice constant parameters, (2) members where m = 1 belong to centrosymmetric nonpolar space groups which contain an inversion center, and (3) members where m = 2 belong to noncentrosymmetric polar space which do not contain an inversion center.

**Chemical Formula**	**"A" site cation CN**	***c* (Å)**	**Space group**	**Polar or Nonpolar**	m
Cu_2Ta4O_{11}	2	0.0	$R\bar{3}c$	Nonpolar	1
$Ag_2Nb_4O_{11}$	6	37.0163	$R\bar{3}c$	Nonpolar	1
$Ag_2Ta_4O_{11}$	6	36.86	$R\bar{3}c$	Nonpolar	1
$Na_2Ta_4O_{11}$	7	36.56	$R\bar{3}c$	Nonpolar	1
$PbTa_4O_{11}$	7	36.8613	*R3*	Polar	2
$Na_2Nb_4O_{11}$	7	12.745	*C2/c*	Nonpolar	1
$CaTa_4O_{11}$	8	12.47	$P6_322$	Polar	2
$SrTa_4O_{11}$	8	12.33	$P6_322$	Polar	2

Table S3. A table of selected members comprised of single and double pentagonal bipyramid layers alternating with octahedral layers. The space group, unit cell parameters, and interatomic distances listed for symmetry unique MO_7 pentagonal bipyramids (M = Nb, Ta) are included.

Layer	Chemical Formula	Space Group	*a* (Å)	*b* (Å)	*c* (Å)	Interatomic distances (Å)	Ref.
**Alternating single and double**	$Cu_5Ta_{11}O_{30}$	$P\bar{6}2c$	6.2297(2)	6.2297(2)	32.550(2)	TaO_7: Ta1-O 1.975(4) x2<br>Ta1-O 1.999(6) x2<br>Ta1-O 2.020 (2) x2<br>Ta1-O 2.422 (8)<br>Ta2-O 1.898(1)<br>Ta2-O 1.994(7)<br>Ta2-O 2.005(6)<br>Ta2-O 2.012(1)<br>Ta2-O 2.013(7)<br>Ta2-O 2.048(4)<br>Ta2-O 2.413(5)	[44,47]
	$Cu_7Ta_{15}O_{41}$	$P6_3/m$	6.2262	6.2262	44.877	N/A	[40]
	$Pr_2Nb_{11}O_{30}$	$P\bar{6}2c$	6.2325(5)	6.2325(5)	32.3677(36)	NbO_7: Nb1-O 1.947 x2<br>Nb1-O 1.967 x2<br>Nb1-O 2.060 x2<br>Nb1-O 2.488<br>Nb2-O 1.894<br>Nb2-O 1.995<br>Nb2-O 2.002<br>Nb2-O 2.005<br>Nb2-O 2.067<br>Nb2-O 2.084<br>Nb2-O 2.424	[49]

Table S4. A table of selected members comprised of double pentagonal bipyramid layers alternating with octahedral layers. The space group, unit cell parameters, and interatomic distances listed for symmetry unique MO_7 pentagonal bipyramids (M= Nb, Ta) are included.

Layer	Chemical Formula	Space Group	*a* (Å)	*b* (Å)	*c* (Å)	Interatomic distances (Å)	Ref.
n=2, double	$BiTa_7O_{19}$	$P\bar{6}c2$	6.2197(2)	6.2197(2)	20.02981(9)	TaO_7: Ta-O 1.9064(8)<br>Ta-O 1.977(3)<br>Ta-O 1.990(3)<br>Ta-O 2.002(1)<br>Ta-O 2.049(2)<br>Ta-O 2.024(1)<br>Ta-O 2.4415(9)	[35]
	$Cu_3Ta_7O_{19}$	$P6_3/m$	6.2278(1)	6.2278(1)	20.1467(3)	TaO_7: Ta-O 1.895(3)<br>Ta-O 1.979(6)<br>Ta-O 1.984(1)<br>Ta-O 2.016(2)<br>Ta-O 2.068(4)<br>Ta-O 2.071(3)<br>Ta-O 2.508(5)	[47]
	$CeTa_7O_{19}$	$P\bar{6}c2$	6.226(3)	6.226(3)	19.976(8)	TaO_7: Ta-O 1.886(6)<br>Ta-O 1.97(4)<br>Ta-O 1.981(6) x2<br>Ta-O 2.06(2) x2<br>Ta-O 2.48(3)	[50,56]
	$EuTa_7O_{19}$	$P6_3/mcm$	6.217	6.217	19.9	N/A	[46]
	$DyTa_7O_{19}$	$P\bar{6}c2$	6.199(3)	6.199(3)	19.859(6)	TaO_7: Ta-O 1.87(2)<br>Ta-O 1.891(3)<br>Ta-O 2.04(2)<br>Ta-O 2.011(3)<br>Ta-O 2.07(1)<br>Ta-O 2.09(2)<br>Ta-O 2.45(1)	[51]
	$LaTa_7O_{19}$	$P\bar{6}c2$	6.23(6)	6.23(6)	19.99(5)	TaO_7: Ta-O 1.896(1)<br>Ta-O 1.926(1)<br>Ta-O 1.940(1)<br>Ta-O 1.997(1)<br>Ta-O 2.046(1)<br>Ta-O 2.094(1)<br>Ta-O 2.463(1)	[52]
	$NdTa_7O_{19}$	$P6_3/mcm$	6.225(2)	6.225(2)	19.94(2)	N/A	[45,58]
	YTa_7O_{19}	Undetermined	6.2035(2)	6.2035(2)	19.865(1)	N/A	[45,58]

Table S5. A table of selected members comprised of single and triple pentagonal bipyramid layers alternating with octahedral layers. The space group, unit cell parameters, and interatomic distances listed for symmetry unique MO_7 pentagonal bipyramids (M= Nb, Ta) are included.

Layer	Chemical Formula	Space Group	*a* (Å)	*b* (Å)	*c* (Å)	Interatomic distances (Å)	Ref.
**n=2, single and triple**	$CeNb_7O_{19}$	*P3*	6.2481(3)	6.2481(3)	20.0566(11)	N/A	[56]
	$LaNb_7O_{19}$	*P3*	6.2531(2)	6.2531(2)	20.0685(10)	NbO_7: Nb1-O 1.945<br>Nb1-O 1.954<br>Nb1-O 1.969<br>Nb1-O 1.974<br>Nb1-O 2.065<br>Nb1-O 2.068<br>Nb1-O 2.496<br>Nb2-O 1.837<br>Nb2-O 2.004<br>Nb2-O 2.007<br>Nb2-O 2.010<br>Nb2-O 2.075<br>Nb2-O 2.109<br>Nb2-O 2.436<br>Nb3-O 1.963<br>Nb3-O 1.968<br>Nb3-O 2.006 x2<br>Nb3-O 2.011<br>Nb3-O 2.022<br>Nb3-O 2.413<br>Nb4-O 1.842<br>Nb4-O 2.003<br>Nb4-O 2.006<br>Nb4-O 2.012<br>Nb4-O 2.071<br>Nb4-O 2.100<br>Nb4-O 2.440	[57]
	$NdNb_7O_{19}$	*P3*	6.2365(2)	6.2365(2)	20.0183(10)	N/A	[45,58]
	$PrNb_7O_{19}$	*P3*	6.2455(3)	6.2455(3)	20.5017(11)	N/A	[45,58]
